# Single-Phase Lithiation in Iron Hydroxy Fluorides with Pyrochlore Structure

**DOI:** 10.1021/acsenergylett.5c00218

**Published:** 2025-02-06

**Authors:** Julian F. Baumgärtner, Dragos C. Stoian, Kenneth P. Marshall, Mohammad Jafarpour, Matthias Klimpel, Huanyu Zhang, Faruk Okur, Wouter van Beek, Dmitry Chernyshov, Sina Abdolhosseinzadeh, Michael Wörle, Maksym V. Kovalenko, Kostiantyn V. Kravchyk

**Affiliations:** † Laboratory of Inorganic Chemistry, Department of Chemistry and Applied Biosciences, ETH Zürich, CH-8093 Zürich, Switzerland; ^‡^Laboratory for Thin Films and Photovoltaics, ^⊥^Laboratory for Functional Polymers, Empa - Swiss Federal Laboratories for Materials Science & Technology, CH-8600 Dübendorf, Switzerland; § Swiss−Norwegian Beam Lines at the European Synchrotron Radiation Facility, Grenoble 38000, France; ∥ Institute of Materials Science and Engineering, Swiss Federal Institute of Technology Lausanne, CH-1015 Lausanne, Switzerland

## Abstract

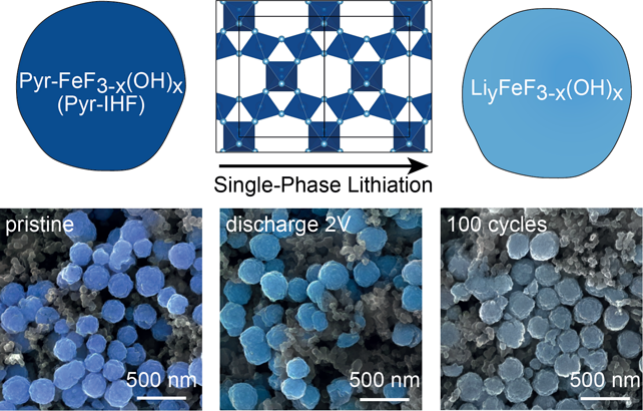

3D transition metal fluorides have long been recognized as appealing low-cost, high-energy-density cathode materials for Li-ion batteries, but their conversion-type lithiation mechanism induces structural and morphological changes, limiting their cycling stability. Our findings now suggest that metal fluorides may undergo single-phase lithiation when crystallized in a pyrochlore structure, enabled by the presence of Li-ion storage sites within interconnected hexagonal channels. By conducting a detailed analysis of pyrochlore iron(III) hydroxy fluorides during lithiation using *operando* X-ray absorption spectroscopy, X-ray total scattering, and electron microscopy, we provide evidence for a possible single-phase lithiation mechanism and robust structural stability. These results challenge the traditional view of conversion-type lithiation in metal fluorides and highlight their potential for achieving high cycling stability and eventual commercialization in Li-ion batteries.

Over the last two decades, the need to further reduce lithium-ion battery costs has driven a surge in reports focusing on low-cost cathodes.^1−4^ In the quest for economically viable alternatives to cathodes containing cobalt and nickel, such as LiCoO_2_ (LCO) or LiNi_*x*_Mn_*y*_Co_1–*x*–*y*_O_2_ (NMC), iron fluorides emerge as compelling active materials. Comprised of earth-abundant elements, they offer a high lithiation potential of 2.7–3.1 V vs. Li^+^/Li, coupled with a substantial theoretical capacity of ca. 237 mA h g^–1^ for one-electron operation.^5−8^

However, commercialization of iron fluorides is hindered by their low cycling stability, attributed to the conversion-type lithiation mechanism, which is believed to be inherent to iron fluorides.^9^ The associated phase transformations cause volume changes and pulverization of the active phase, causing contact loss with the conductive network, and ultimately capacity fade (Figure 1a).^10,11^ At the heart of this problem is the lack of available Li-ion storage sites within the crystal structure of rhombohedral FeF_3_ (r-FeF_3_), which adopts a distorted ReO_3_ structure (*R*3̅*c*).^12^ While initial topotactic lithiation of r-FeF_3_via a single Li_*x*_FeF_3_ phase has been reported,^13,14^ such intermediate Li_*x*_FeF_3_ phases ultimately convert to FeF_2_ and LiF, leading to capacity fade (Figure 1a). Conceptually, the required Li-ion storage sites can be introduced into the r-FeF_3_ structure, by removing every second FeF_3_ unit along the (1–10) or equivalent directions, followed by a translation of the Fe atoms in the resulting porous layer along 1/2 (−1–10), and rotation by 90° around (110) to yield the cubic pyrochlore structure (Figure 1a,b).^15^ While the electrochemical performance of pyrochlore iron fluorides,^16−20^ and other porous iron fluoride modifications has been reported,^21−24^ validating the potential single-phase lithiation, is obstructed by rapid amorphization even upon the initial lithiation cycle.^16,18,24,25^ We note that the term “single-phase lithiation” is defined herein following the work of Hua et al.,^13^ and refers to a lithiation mechanism with a single lithiated phase, in contrast to conversion mechanisms, which involve the formation of multiple phases.

**Figure 1:**
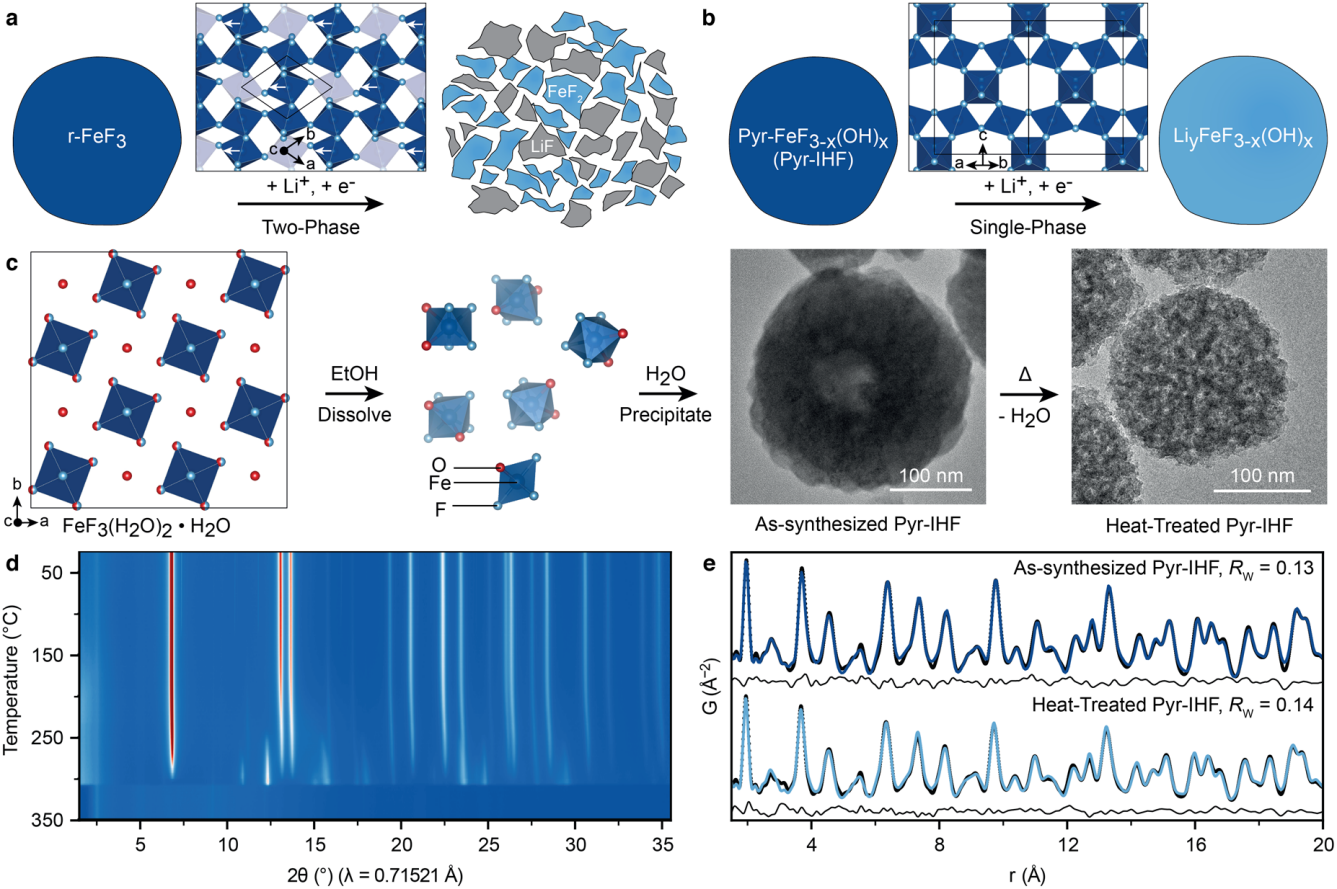
Proposed lithiation mechanism and synthesis of pyrochlore iron hydroxy fluoride (Pyr-IHF). (a, b) Illustration of structural changes during lithiation of FeF_3_ active material particles with (a) ReO_3_ structure or (b) pyrochlore structure. The structural relationship between the r-FeF_3_ structure and the pyrochlore structure is highlighted in (a) by showing the Fe(F/OH)_6_ octahedra that need to be removed from the r-FeF_3_ structure, followed by adequate repositioning of the remaining octahedra to result in the pyrochlore structure. (c) Synthetic scheme for Pyr-IHF. FeF_3_(H_2_O)_2_·H_2_O partially dissolves in EtOH, and after centrifugation and H_2_O addition to the ethanolic solution, as-synthesized Pyr-IHF precipitates. (d) *In situ* synchrotron XRD of as-synthesized Pyr-IHF during heat treatment. (e) PDF refinements for as-synthesized Pyr-IHF (dark blue) and heat-treated Pyr-IHF (light blue) in the low-r region (black dots, observed; colored line, calculated; black line, difference).

In this study, we challenge the generalized view of iron fluoride as solely conversion-type cathode materials by examining the phase changes in pyrochlore iron hydroxy fluoride (Pyr-IHF) during lithiation through comprehensive investigations utilizing *operando* X-ray absorption spectroscopy (XAS), synchrotron X-ray total scattering, and electron microscopy. We provide compelling evidence for the robust structural stability of Pyr-IHF during one-electron operation. Our findings indicate that the interconnected 3D channels of Pyr-IHF not only facilitate Li-ion diffusion, as previously reported,^16−18^ but may also support single-phase lithiation, fundamentally distinct from the conversion mechanism observed in r-FeF_3_, thereby significantly enhancing particle stability and mitigating capacity degradation. Our research showcases the remarkable cycling stability of Pyr-IHF, sustaining 500 cycles at a current density of 100 mA g^–1^, coupled with a substantial cumulative discharge capacity nearing 60000 mA h g^–1^.

## Synthesis of Pyrochlore Iron Hydroxy Fluoride (Pyr-IHF)

Pyr-IHF was synthesized according to our previous report.^17^ First, FeF_3_(H_2_O)_2_·H_2_O precursor was partially dissolved in ethanol (Figure 1c), with water then injected into the resulting ethanolic solution to precipitate as-synthesized Pyr-IHF. Upon addition of H_2_O, the dissolved Fe(F/OH)_6_ octahedra condensate together with H_2_O acting as a templating agent, to yield the cubic pyrochlore structure (*Fd*3̅*m*). The structure and purity of as-synthesized Pyr-IHF were verified by Rietveld refinement (Figure S1, Table S1). The lattice constant of 10.42162(14) Å matches that of previously obtained Pyr-IHF after vacuum drying.^17^ Zeolithic H_2_O (0.6 per formula unit) occupies the 8c site, and partially the 16d site within the channels of Pyr-IHF. Noteworthy, the obtained product contained less than 2 wt % of FeF_3_(H_2_O)_2_·H_2_O impurities, significantly less than in the previously reported synthesis. The higher purity is attributed to an additional washing step before drying. Spherical particles of as-synthesized Pyr-IHF with a narrow particle size distribution were observed by transmission electron microscopy (Figure 1c, Figure S2). Finally, the optimal temperature to remove zeolithic H_2_O molecules from as-synthesized Pyr-IHF was determined to be between 240 to 275 °C, referred to subsequently as heat-treated Pyr-IHF, using *in situ* synchrotron X-ray diffraction (Figure 1d, Figures S5–S7, Table S5, Supplementary Text).

Heat-treated Pyr-IHF had a significantly contracted unit cell parameter compared to as-synthesized Pyr-IHF (10.36 Å vs. 10.42 Å), and contained 0.4 H_2_O per formula unit, as determined by Rietveld refinement (Figure S1, Table S2). Dehydration during heat treatment induces partial amorphization within Pyr-IHF (20–40 wt %).^17^ To validate if the characteristic channels of the pyrochlore structure are retained in the partially amorphized sample, synchrotron X-ray total scattering was measured for as-synthesized and heat-treated Pyr-IHF (Figure 1e, Figures S3–S4, Tables S3–S4). The pair distribution functions (PDF) in the low-*r* region (1.5–20 Å) confirmed the locally preserved pyrochlore phase after heat treatment (R_w_ of 13.0% and 13.8%, respectively), alongside minor rutile FeOF impurities (10 wt %). Notably, in addition to the crystallographic changes, heat treatment of as-synthesized Pyr-IHF also altered the Pyr-IHF nanoparticle morphology, increasing their porosity but retaining their overall size and shape (Figure 1c, Figure S2).

## Stability of Pyr-IHF Particles during Electrochemical Cycling

**Figure 2:**
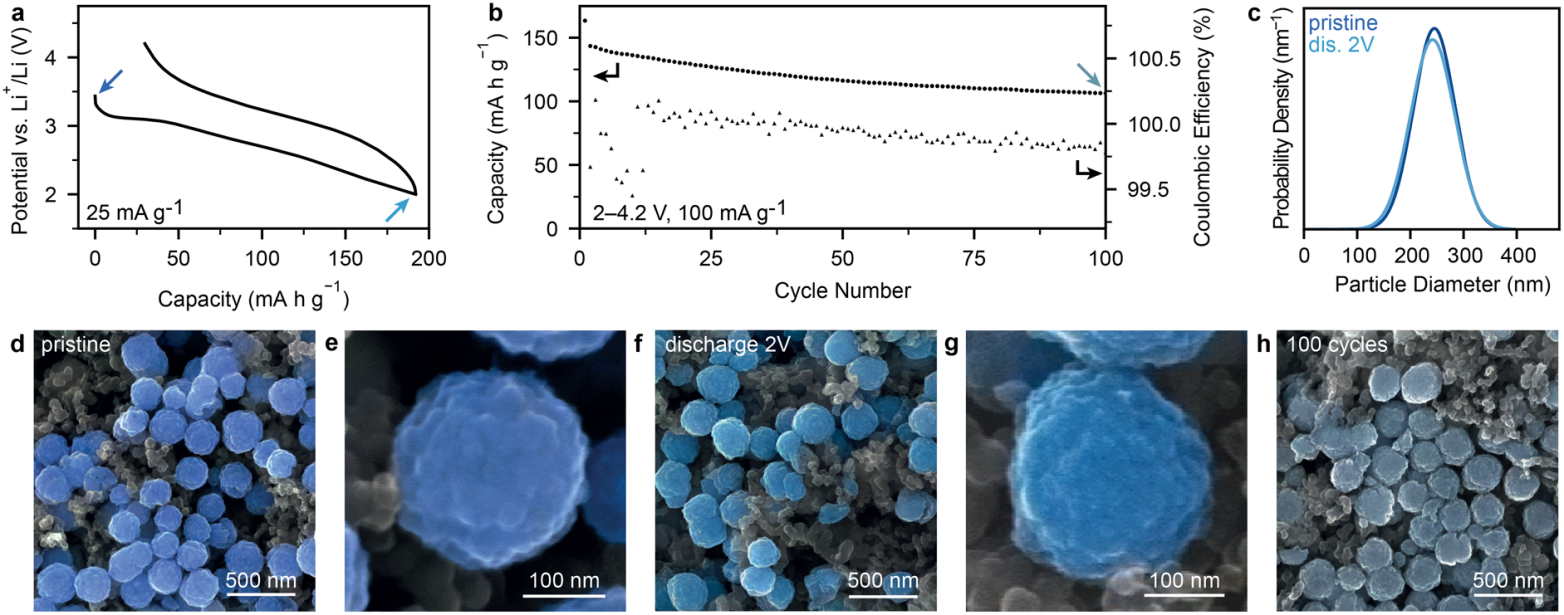
Stability of Pyr-IHF particles during electrochemical cycling. (a) Voltage profile of full cells with Pyr-IHF cathodes during the first cycle. Colored arrows denote points at which scanning electron microscopy (SEM) micrographs were obtained. (b) Cycling stability of full cells with Pyr-IHF cathodes. (c) Particle size distribution of Pyr-IHF particles in cathodes after preparation and after discharge to 2 V. (d, e) SEM micrographs of pristine Pyr-IHF cathodes. Colored particles indicate Pyr-IHF active material particles, and gray parts represent carbon black. (f, g) SEM micrographs of Pyr-IHF cathodes after discharge to 2 V. (h) SEM micrograph of Pyr-IHF cathodes after 100 cycles.

Figure 2a displays the initial voltage profile of coin cells with Pyr-IHF cathodes prepared in air and Li metal anodes using a Li-ion conducting ionic liquid electrolyte at a current density of 25 mA g^–1^ (ca. C/10) within 2–4.2 V. While the initial discharge capacity was 192 mA h g^–1^, 162 mAh g^–1^ could be extracted during recharge, indicating an initial capacity loss of ca. 30 mA h g^–1^ and a Coulombic efficiency of 118%. Cycling stability measurements of Pyr-IHF cathodes at a higher current density of 100 mA g^–1^ (ca. 0.4 C) showed a similarly high initial discharge capacity of 164 mA h g^–1^, along with a capacity retention of 74% after 100 cycles, and an exceptional Coulombic efficiency of 99.92%, excluding the first cycle capacity loss (Figure 2b).

This initial capacity loss in pyrochlore iron fluorides has been documented in previous reports.^16−18^ Such a capacity loss commonly occurs in iron fluorides due to phase transformations during lithiation, causing contact loss with the conductive network.^26,27^ Remarkably, Pyr-IHF-containing cathodes exhibited exceptional structural stability of the active material particles during cycling, as evidenced by scanning electron microscopy (SEM) of pristine and cycled cathodes (Figure 2d–h. Figures S9–S11, Tables S7–S8). Pyr-IHF particles prior to cycling have a very narrow particle size distribution (standard deviation of 40 nm) around a mean diameter of 245 nm, and are well approximated by a normal or Weibull distribution (Figure 2c, Figure S10–S12, Table S7). This stands in stark contrast with the broader log-normal particle size distributions used to describe active materials, including iron fluorides.^28,29^ After discharge to 2 V, the particle size distribution remains nearly identical with a mean particle diameter of 240 nm and a standard deviation of 40 nm, evidencing the excellent structural integrity of the discharged Pyr-IHF particles with minimal volume change compared to the pristine state (Figure 2f,g, Figures S11–S12, Table S8). Moreover, even after 100 cycles, the particle size distribution and morphology remain intact, demonstrating the unexpectedly high structural stability of Pyr-IHF particles during cycling (Figure 2h, Figure S13).

In contrast, conversion-type r-FeF_3_ particles undergo significant structural changes and pulverization, even with very small active material particles (10–50 nm).^30^ The exceptional structural stability of Pyr-IHF particles during cycling contradicts a conversion-type mechanism involving the formation of multiple phases during lithiation. Instead, the sustained structural integrity of Pyr-IHF during cycling suggests that a single-phase mechanism is more plausible.

## Probing the Origin of Structural Stability in Pyr-IHF

**Figure 3:**
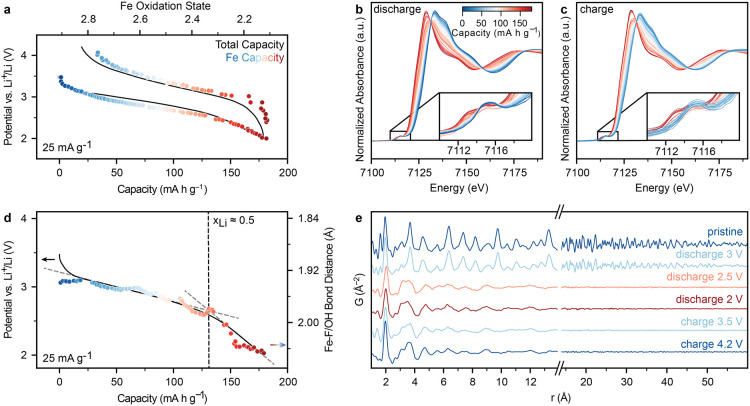
Structural characterization of Pyr-IHF during electrochemical cycling. (a) Voltage profiles of a full cell with Pyr-IHF cathodes obtained by electrochemistry (black) and from the Fe capacity contribution determined by Fe oxidation state changes according to MCR analysis (blue/red). (b, c) Normalized *operando* XANES spectra at the Fe K-edge during (b) first discharge and (c) first charge with the pre-edge magnified. Color corresponds to the electrochemical capacity. (d) Evolution of the Fe–F/OH bond distance in Pyr-IHF during lithiation according to EXAFS fitting (blue/red), and corresponding voltage profile (black) (see also Figure S17). (e) *Ex-situ* PDFs of full cells with Pyr-IHF cathodes at different states of charge of the first cycle.

To elucidate the underlying reason for the high structural stability of Pyr-IHF, cathodes were characterized during lithiation by *operando* XAS (Figure 3a–d, Figure S14). X-ray absorption near edge spectra (XANES) were examined to determine oxidation state changes of Fe in Pyr-IHF. During initial discharge, the edge energy decreased, reflecting the expected Fe^+III^ to Fe^+II^ reduction (Figure 3b). Upon charging, the edge energy increased, indicating Fe oxidation. Notably, the original spectrum of pristine Pyr-IHF was not fully recovered (Figure 3c), indicating partially irreversible lithiation of Pyr-IHF.

To exclude other capacity contributions, apart from that of Pyr-IHF, the Fe oxidation state and the associated charge storage capacity were quantified using principal component analysis (PCA) and multivariate curve resolution (MCR) of the *operando* XANES spectra (Figure 3a, Figures S15 and S16, Supplementary Text). The capacity originating exclusively from Fe reduction/oxidation, as determined by MCR closely matched the electrochemically determined capacity (179 mA h g^–1^ vs 181 mA h g^–1^ after full discharge). After charge, 77% of Fe^+III^ was recovered, corresponding to 149 mA h g^–1^, consistent with the reduced electrochemical charge capacity of 145 mA h g^–1^. Such close agreement between MCR-calculated and electrochemical capacity excludes parasitic side reactions, e.g., electrolyte degradation,^9^ as a potential source for the initial capacity loss.

Iron fluorides frequently display concurrent reduction of both Fe^+III^/Fe^+II^ and Fe^+II^/Fe^0^ above 2 V.^31^ Since only the Fe^+III^/Fe^+II^ couple may proceed via single-phase lithiation, separating the two reduction processes into distinct voltage regimes associated with Fe^+III^/Fe^+II^ or Fe^+II^/Fe^0^ reduction is necessary to prevent phase transformations. In contrast to r-FeF_3_,^31^ no Fe^0^ formed in Pyr-IHF cathodes during cycling, as evidenced by PCA, MCR and the preserved pre-edge intensity during cycling (Figure 3b,c, Figures S15–S16). The distinct voltage plateaus in Pyr-IHF cathodes likely stem from the narrow particle size distribution of Pyr-IHF, coupled with fast lithiation kinetics for Fe^+III^/Fe^+II^ reduction. In contrast, other metal fluorides often display broad particle size distributions depending on the synthesis. Combined with sluggish reaction kinetics, the overpotential for lithiation of r-FeF_3_ therefore strongly depends on particle size.^9^ Consequently, the nanosized or surface-exposed fraction of r-FeF_3_ active material may be reduced to Fe^0^ while larger particles still contain Fe^+III^.

Interestingly, despite exclusive Fe^+III^/Fe^+II^ reduction in Pyr-IHF during lithiation, its initial discharge voltage profile exhibits two distinct regions: a flat region until ca. 2.7 V (ca. 130 mA h g^–1^), and a sloped regime at lower voltages (Figure 3d, Figure S17). Only 0.5 Li^+^ (ca. 114 mA h g^–1^) can be inserted into the 3D channels of Pyr-IHF without significant Coulombic Li-ion repulsion, which aligns with the observed transition between the flat and sloped voltage regions. We therefore reasoned that the flat profile is linked to lithiation of the Pyrochlore phase, while the larger structural changes within the host structure required to accommodate further Li-ions give rise to a sloped voltage profile.

To verify this hypothesis, the structural changes in Pyr-IHF were probed on an atomic level using *operando* extended X-ray absorption fine structure (EXAFS) (Figure S18). Interestingly, the Fe–F/OH bond distance from first-shell EXAFS fitting increased marginally in the flat voltage region, before elongating significantly beyond 130 mA h g^–1^, thereby closely mirroring the voltage profile trends (Figure 3d).

Additional *ex-situ* PDF of cathodes discharged and recharged to different potentials during the initial cycle confirmed that the Pyr-IHF structure is retained until discharge to 3 V (Figure 3e, Figure S19). PDF fitting indicated a remarkably small volume expansion of 0.6 vol % during lithiation with the lattice constant increasing from 10.403 Å before lithiation to 10.424 Å for Pyr-IHF discharged to 3 V (Figures S25). Notably, only a single phase was required to fit the PDF of Pyr-IHF discharged to 3 V, indicating solid solution lithiation akin to LCO and NMC.^32^ Solid-solution lithiation is further supported by the absence of sharply defined isosbestic points in the *operando* XANES spectra. Upon further discharge to 2.5 V, the Pyrochlore structure is amorphized, losing its medium-range and long-range order (Figure S21). However, the amorphized phase retains the low density of the pyrochlore parent phase, and PDF fitting also suggests that the hexagonal channels as the local structural motif may be preserved, consistent with a single-phase lithiation mechanism (Figure S26, TableS9, Supplementary Text).

## Electrochemical Performance of Pyr-IHF with Optimized Cathode Architecture

Although the volume of the ca. 250 nm large particles remained unchanged upon cycling, lithiation and the subsequent amorphization may induce volume change within the individual domains of the porous particle. As a result, the individual domains may lose contact with each other, leading to macroscopic particle cracking, as evidenced by SEM micrographs of cathodes after complete discharge (Figure 2, Figures S27–S31). After 100 cycles, even more particles exhibited cracking, suggesting that lithiation-induced stress cracking persists beyond the initial cycle (Figures S30–S31). Cracking may cause the active material particles to delaminate from the conductive network, resulting in the observed capacity loss.

**Figure 4:**
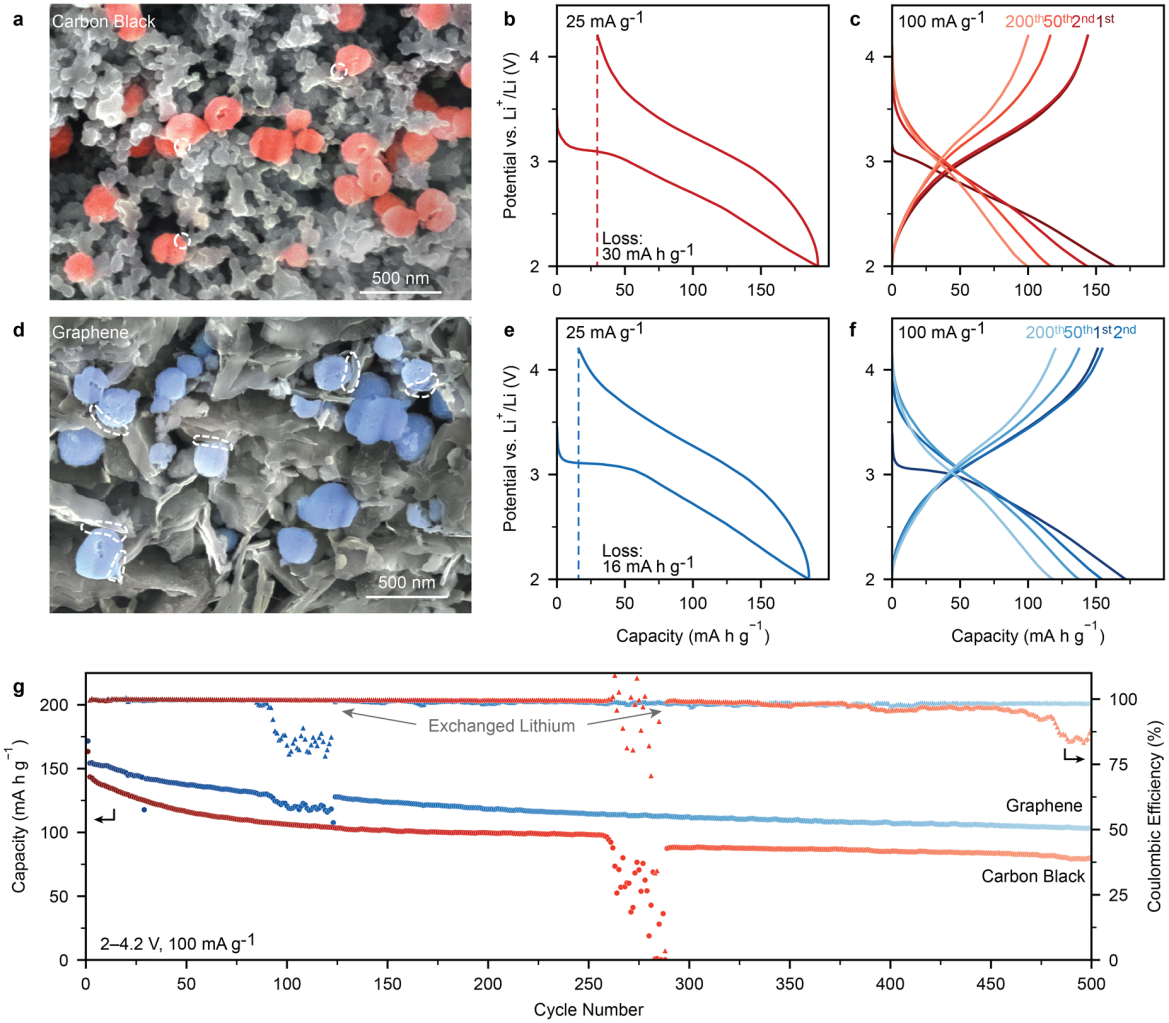
Electrochemical performance of Pyr-IHF with optimized cathode architecture. (a, d) Cross-sectional SEM micrographs of pristine Pyr-IHF cathodes containing (a) carbon black (CB) (d) and graphene conductive additive. Dashed circles indicate contact areas between conductive additive and Pyr-IHF. (b, e) Voltage profile of full cells with Pyr-IHF cathodes containing (b) CB and (e) graphene conductive additives during the first cycle. (c, f) Voltage profile of full cells with Pyr-IHF cathodes containing (c) CB and (f) graphene conductive additives for selected cycles. (g) Cycling stability of the cathodes shown in (c, f). Gray arrows indicate the cycles at which spent Li metal anodes were exchanged for fresh ones.

Recognizing the importance of mitigating contact loss, we reasoned that an increased contact area between the conductive network and the active material particles could address this issue. While carbon black (CB) is a common conductive additive, its spherical nature only allows for point-to-point contacts with the active material.^10^ In contrast, high surface area conductive additives based on 2D materials like graphene offer the potential for more effective plane-to-point contacts, which can be maintained throughout cycling.^10,33^ Hence, Pyr-IHF cathodes were fabricated with a graphene ink developed in our lab.^34^ Unlike conventional reduced graphene oxide, these pristine graphene inks are distinguished by high degrees of graphitization and low amounts of heteroatom impurities, ensuring high electronic conductivity. SEM confirmed the large contact area between Pyr-IHF particles and graphene in the prepared cathodes (Figure 4a, d).

Pyr-IHF cathodes with graphene demonstrated significantly enhanced electrochemical performance compared to those with CB additives. While Pyr-IHF cathodes with CB exhibited discharge and charge capacities of 192 mA h g^–1^ and 162 mA h g^–1^, respectively, with a 30 mA h g^–1^ capacity loss, cathodes containing graphene achieved a similarly high discharge capacity of 185 mA h g^–1^, but could retain 170 mA h g^–1^ during recharge, a ca. 50% reduction in capacity loss (Figure 4b,e). Cycling stability measurements at 100 mA g^–1^ (ca. 0.4 C) demonstrated similarly improved cycling stability for graphene-containing Pyr-IHF cathodes (Figure 4c,f,g). Excluding the first cycle capacity loss, CB-containing electrodes exhibited a discharge capacity retention of 81%, 69% and 56% after 50, 200, and 500 cycles, respectively. Meanwhile, graphene-containing electrodes demonstrated capacity retentions of 89%, 77% and 67% after 50, 200, and 500 cycles. The cumulative discharge capacity over these cycles (59,000 mA h g^–1^ over a cycle life of 1180 h, ca. 50 days) ranks among the highest reported ones for iron (oxy/hydroxy) fluorides (Figure S32).

In conclusion, this study explored the feasibility of single-phase lithiation in metal fluorides with a suitable crystal structure and provided supporting evidence for this mechanism. Our findings demonstrate that metal fluorides with a pyrochlore structure bypass the structural instability and capacity fade typically associated with conversion-type metal fluorides. The high structural stability is consistent with a single-phase lithiation process, and enables highly stable cycling performance. This work paves the way for developing low-cost metal fluoride cathodes with high cycling stability, a crucial step toward their commercial viability.

## Data Availability

All data are available in the main text or the Supporting Information, and available from the corresponding author upon reasonable request.
