# Supplementary material for: Single-Phase Lithiation in Iron Hydroxy Fluorides with Pyrochlore Structure
Source: ACS Energy Lett. 2025 Feb 6;10(2):1082–8. doi: 10.1021/acsenergylett.5c00218 (PMC11833864; doi:10.1021/acsenergylett.5c00218)
Supplement: Supplementary file 1 — nz5c00218_si_001.pdf [file nz5c00218_si_001.pdf]

Supplementary Materials for

Single-Phase Lithiation in Iron Hydroxy Fluorides with Pyrochlore Structure

Julian F. Baumgärtner,[1,2] Dragos C. Stoian,[3] Kenneth P. Marshall,[3] Mohammad Jafarpour,[2,4] Matthias Klimpel,[1,2] Huanyu Zhang,[1,2] Faruk Okur,[1,2] Wouter van Beek,[3] Dmitry Chernyshov,[3] Sina Abdolhosseinzadeh,[2] Michael Wörle,[1] Maksym V. Kovalenko,[1,2] and Kostiantyn V. Kravchyk[1,2*]*

[1]Laboratory of Inorganic Chemistry, Department of Chemistry and Applied Biosciences, ETH Zürich, CH-8093 Zürich, Switzerland.

[2]Laboratory for Thin Films and Photovoltaics, Empa - Swiss Federal Laboratories for Materials Science & Technology, CH-8600 Dübendorf, Switzerland.

[3]Swiss–Norwegian Beam Lines at the European Synchrotron Radiation Facility, 38000 Grenoble, France.

[4]Institute of Materials Science and Engineering, Swiss Federal Institute of Technology Lausanne, CH-1015 Lausanne, Switzerland.

*Corresponding authors. Emails: Kostiantyn.Kravchyk@empa.ch and mvkovalenko@ethz.ch

EXPERIMENTAL METHODS

Synthesis of Pyr-IHF

The synthesis of Pyr-IHF has been described previously.[1] $FeF_3(H_2O)_2 \cdot H_2O$ (500 mg, 3.00 mmol, abcr chemicals) was mixed with EtOH (150 mL, absolute for analysis, 99.8%, EMSURE, ACS) in a 250 mL capped bottle. The resulting suspension was stirred vigorously for 6 to 9 hours at room temperature (RT) to partially dissolve $FeF_3(H_2O)_2 \cdot H_2O$, forming an iron hydroxy fluoride ethanolic solution (*ca.* 1 g_{Fe} L_{EtOH}^{-1}) The suspension was centrifuged (10000 rpm, 5 min) and the clear yellow solution was decanted with a syringe to separate it from the undissolved $FeF_3(H_2O)_2 \cdot H_2O$. The solution was then filtered through a PTFE syringe filter (0.45 µm). Subsequently, 6 mL (4 vol.-%) of distilled water was added to the solution and allowed to precipitate for 24 h. The suspension was then centrifuged (10000 rpm, 5 min), and the residual beige powder of Pyr-IHF was washed with EtOH. Pyr-IHF was then dried under vacuum at 80 ˚C overnight. The weight of the dried Pyr-IHF powder was 103(23) mg (0.86(19) mmol (standard deviation in brackets, based on 6 experiments).

Heat Treatment of Pyr-IHF

The heat-treatment of Pyr-IHF has been described previously.[1] As-synthesized Pyr-IHF (*ca.* 100 mg) was placed in a 2 mL glass vial in a tube furnace (Carbolite Gero) and the sides were sealed with quartz wool. Pyr-IHF was heated to 240 to 275 ˚C for 30 min in air at a heating rate of

10 °C min^{-1}, followed by a natural cooling rate by switching off the oven. The resulting Pyr-IHF powders were orange-brown in color.

Preparation of Graphene Inks

The preparation of graphene inks has been described previously.[2] Graphite powder was added to NMP with an initial concentration of 50 mg mL^{-1}, and sonicated for 80 h in a bath sonicator (BANDELIN SONOREX SUPER, 600 W, 35 kHz). The obtained suspension was then centrifuged at 4500 rcf for 30 min, and the supernatant was collected. In a separation funnel, 400 ml of xylene was added to and mixed with 1 L of the obtained supernatant. By adding 600 mL of deionized water, an emulsion was formed, which separated into a three-phase system. The middle phase containing graphene concentrate was collected and washed first with ethanol and then with NMP using centrifugation and redispersion. The final ink solution contained 6.12(20) wt.-% graphene.

Battery components

Carbon black (CB, Super C65, TIMCAL), a glass-microfiber separator (Whatman), Poly(vinylidene fluoride) (pVdF, average Mw ~534 g mol^{-1}, Sigma Aldrich), and N-Methyl-2-pyrrolidone (NMP, 99%, Sigma Aldrich), Lithium bis(trifluoromethanesulfonyl)imide (LiTFSI, 99+%, Solvionic), 1-Butyl-1-methylpyrrolidinium bis(trifluoromethylsulfonyl)imide ($Pyr_{1,4}TFSI$, 99%, iolitec, dried over molecular sieves prior to use).

Synchrotron Powder X-ray Diffraction

SXRD data were obtained at the BM01 beamline at the European Synchrotron Radiation Facility. The X-ray beam (λ = 0.71521 Å) was tuned with a sagitally focusing Si(111) double crystal monochromator and a set of collimating and vertically Rh-coated Si mirrors to a final beam size of *ca.* 0.1 mm x 0.35 mm. Diffraction patterns were collected in transmission mode on a Pilatus 2M area photon-counting detectors, and azimuthally integrated using BUBBLE.[3] Patterns were acquired for 5 sec in a 2θ range of 3 – 35 ° with a step size of 0.011 ° at RT. Samples were prepared in a 0.5 mm Ø borosilicate glass capillary and sealed under air.

For the variable-temperature SXRD, the sample was heated under air to 350 °C at 10 K min^{-1} and cooled down again at 10 K min^{-1} using a Cryostream 700+.

For the Rietveld refinement on SXRD data, instrumental parameters were determined with a LaB_6 NIST standard. Rietveld refinement was performed with the GSAS-II program.[4] The background was modelled by a Chebyschev polynomial with 20 coefficients. The refined values for as-synthesized and heat-treated Pyr-IHF are summarized in Tables S1 and S2.

Synchrotron X-Ray Total Scattering

X-ray total scattering data was obtained at the BM31 beamline at the European Synchrotron Radiation Facility. The X-ray beam (λ = 0.25579 Å) was monochromatized using a liquid nitrogen double crystal monochromator equipped with a flat and a fixed radius sagital Si(111)crystal a final beam size of ca. 0.3 mm x 0.06 mm. Diffraction patterns were collected in transmission mode on a CdTe Pilatus 2M area photon-counting detectors, and azimuthally integrated using BUBBLE.[3]

Patterns were acquired for 20 min in a 2θ range of 1.2 – 68 ° (0.5 – 27 $Å^{-1}$) with a step size of 0.013 ° at RT. Samples were prepared in a 2 mm Ø borosilicate glass capillary and sealed under Ar.

For measurement of the cycled cathodes, the cycled cells were transferred into a glove box (GB) and opened with a decrimping machine. Then, the cathode was scraped off the current collector with a razor blade and transferred into the borosilicate capillary within the GB.

The total scattering data was converted into real space and normalized using PDFgetX3.[5] For as-synthesized and heat-treated Pyr-IHF samples, the full Q-range (0.5 – 27 $Å^{-1}$) was used, and the experimentally determined background signal was subtracted before conversion (Figures S3 and S4, Tables S3 and S4). For the cycled cathodes, a Q-range between 0.5 and 21 to 25 $Å^{-1}$ was used. For background subtraction, a reference cathode including electrolyte, but without active material (Figures S8 and S18 to S23), was measured, and subtracted, to isolate the PDF contribution of the active material. PDF fitting was performed using PDFgui and diffpy-cmi.[6, 7]

X-Ray Absorption Spectroscopy

X-ray absorption data was obtained at the BM31 beamline at the European Synchrotron Radiation Facility. The X-ray beam was monochromatized using a liquid nitrogen double-crystal monochromator equipped with a pair of flat Si(111) crystals. Spectra were recorded in transmission mode at the Fe K-edge. For reference samples of different iron containing compounds (Fe, FeF_2, Fe_2O_3, r-FeF_3, $FeF_3(H_2O)_2 \cdot H_2O$, $FeF_3 \cdot 0.33\ H_2O$, as-synthesized Pyr-IHF, heat-treated Pyr-IHF), spectra were acquired at RT between 7.0 – 8.0 keV, scanning continuously with *ca.*

4 eV s^{-1}, with 3 repeats and a final beam size of *ca.* 6 mm x 5 mm. Reference samples were prepared by grinding *ca.* 12 mg of material with ca. 88 mg of cellulose in a mortar for 5 min. The exact amount was chosen to obtain an edge step of 1.0. The powder was then pressed into 13 mm pellets with a pressing dye. For air-sensitive samples (FeF_2 and r-FeF_3), preparation was done in an Ar-filled GB and the pellet sealed air-tight in a sealing bag. The samples were then stuck onto Kapton tape and mounted onto a sample holder. For the pristine Pyr-IHF-containing cathodes, the 12 mm cathode was folded onto itself multiple times before measurement to maximize signal intensity.

For the operando battery cell, a similar cell design to the one in Ref.[8] was used. To minimize attenuation by the cell, the windows were first sealed with Kapton tape and the Kapton tape was further glued on the sides with epoxy glue. The custom-cell was tested for air-tightness by placing a piece of Li inside and storing the cell outside for two days. A cathode with high loading (1.47 mg cm^{-2}) was prepared (*vide infra*) and soaked with 1 M LiTFSI in Pyr1,4TFSI electrolyte for one week. The microfiber separator was pulled apart to minimize attenuation and a piece of 12 mm Li disk was placed on the opposite side. XAS spectra were obtained every 5 min.

XAS data was analyzed using the Demeter software package,[9] and the Larch python module.[10] Some spectra were excluded from subsequent analysis due to measurement glitches, visible by strong deviations in the EXAFS region (Figure S13). The remaining spectra were averaged over a period of ±12.5 min (±5 mA h g^{-1}) to improve the signal-to-noise ratio. For deglitching, EXAFS data was omitted in the regions of strong deviation, which are mainly related to artifacts from the beamline optics, as seen in Figure S13.

Principal component analysis (PCA) was performed on the *operando* XANES spectra to determine the number of components for subsequent MCR-ALS analysis using the PrestoPronto software package (Figure S15).[11] The number of principal components was chosen according to Kaiser's criterion, only components with variances above 1 were retained.[12]

MCR-ALS analysis was performed using the Matlab code of Tauler *et al.*[13] A two-component system was chosen based on the previous PCA analysis. As the initial guesses for the two components, the XANES spectra for heat-treated Pyr-IHF (Fe^{+III}) and FeF_2 (Fe^{+II}) were used and the components subsequently refined for 16 iterations (Figure S16). Non-negative constraints were applied for both the phase concentration and spectra profiles, and a closure constraint was placed on the concentration profiles (sum of concentrations equal to 1).

EXAFS data were k^2-weighted and the Fourier transform performed in the k-range of 2.7–11 $Å^{-1}$ with a Hanning window, and back-transformed in an R-range 1-2.2 Å with a Hanning window (Figure S17). Fitting of the first-shell Fe-F/OH peak was performed using the Pyr-IHF Fe-F/OH scattering path as an initial guess, and the bond distance, edge energy, Debye-Waller factor, and coordination number as free variables. Because the amplitude reduction factor was not specifically extracted, the coordination numbers were refined relative to the initial spectrum, for which a Fe-F/OH coordination number of 6 was chosen based on the previous Rietveld refinements and PDF fittings.

Electron Microscopy

TEM measurements were performed on a Talos F200X (ThermoFisher Scientific, field emission gun, U_{acc} = 200 kV). Samples were prepared by dispersing Pyr-IHF in ethanol and depositing a few drops of the suspension onto a perforated carbon foil supported on a copper grid.

SEM measurements of Pyr-IHF containing cathodes were recorded on a ZEISS Gemini SEM 460 (10 kV acceleration voltage, 500 pA beam current). A working distance of *ca.* 2.8 mm was used for secondary electron in-lens detection, and *ca.* 8.1 mm for backscattered electron detection.

Cross-section FIB-SEM images were prepared on a Thermo Fisher Scientific Helios 5 Hydra Multi-Ion-Species Plasma FIB Microscope. Cross-sections were made by argon ion beam milling with an acceleration voltage of 30 kV and a current of up to 2.0 μA. Corresponding SEM images were recorded on the ZEISS Gemini SEM 460 (5 kV acceleration voltage, 500 pA beam current, *ca.* 7 mm working distance, *ca.* 52 ° stage tilt).

For measurement of the cycled cathodes, the cells were transferred into a GB and opened with a decrimping machine. Cathodes were then washed in dimethyl carbonate (1 mL) for 24 h, for three times and dried. Cycled cathodes were transferred into the SEM with an inert transfer chamber to avoid contact with air.

To determine the particle size distribution, SEM images were analyzed using ImageJ (Figures S10 and S11).[14] The number of particles used to determine the distribution was 133 for the pristine cathode and 122 after discharge to 2 V. The particle area was determined by equivalent ellipsoids or freely drawn. Next, the radius of the area-equivalent circle was calculated and the particle size distribution was modelled with the reliability module in python,[15] using a maximum likelihood estimation of the distribution parameters (Tables S6 to S8).

Electrochemistry

In a typical cathode preparation, a slurry was prepared from heat-treated Pyr-IHF (100 mg, 50 wt.-%), CB (80 mg, 40 wt.-%) and a solution of 0.833 wt-% pVdF binder in NMP (20 mg, 10 wt.-% pVdF; 2400 mg, 1200 wt.-% NMP). The slurry was mixed under air in a ZrO_2 beaker (12 mL) with ZrO_2 balls (20 g, 5 mm Ø) and ball-milled in a planetary ball-mill (Fritsch, Pulverisette 7) for 1 h at 300 rpm. The slurry was then immediately tape-casted onto carbon-coated Al foil with a doctor blade of 150 μm at a speed of 1 mm s^{-1} to obtain a uniform tape (*ca.* 6 cm x 30 cm). The tape-casted Al foil was dried under air at 125 ˚C until visibly dry (*ca.* 20 min) and then dried under vacuum at 80 ˚C overnight. The tape was then transferred into an Ar-filled GB and 12 mm Ø disks were punched out of the foil and weighed (mean active material loadings with standard deviation was 0.454(12) mg cm^{-2}) inside a GB. The cathode was wetted with 150 μL of Li-ion conducting electrolyte (1 M LiTFSI in $Pyr_{1,4}TFSI$). The ionic liquid electrolyte was chosen because of the good cycling stability obtained in previous studies.[1] Electrolyte-wetted cathodes were then heated under Ar at 75 ˚C overnight to ensure good wetting of the cathode.[16] The cathode was then incorporated with a glass microfiber separator into an air-tight stainless-steel coin-type cell (CR2032, 316L, Hohsen Corp). Elemental Li coins (12mm Ø, *ca.* 100 μm thickness) were used as counter and reference electrodes.

For the cathode used in *operando* XAS measurements, the same procedure was followed but the cathode slurry was tape-casted with a doctor blade of 200 μm. The tape was dried at 80 ˚C and

a subsequent tape-casting with a doctor blade of 200 μm was performed. The cell was then dried as described above. The active material loading was 1.47 mg cm^{-2}.

For the cathodes used for *ex-situ* SPDF measurements, the same procedure was followed, but the cathode slurry was tape-casted with a doctor blade of 300 μm. The active material loading was 1.24(23) mg cm^{-2}.

In a typical cathode preparation for graphene containing electrodes, a slurry was prepared from heat-treated Pyr-IHF (67 mg, 57 wt.-%), and a solution of 6.1 wt-% graphene in NMP (50 mg, 43 wt.-% graphene; 820 mg, 700 wt.-% NMP). The subsequent electrode preparation was done as described in the first paragraph. The final active material loading was 0.76(16) mg cm^{-2}.

Galvanostatic cycling was performed in a voltage range between 2 – 4.2 V on a multichannel potentiostat/galvanostat from Biologic (MPG2) or Astrol BAT-Flex. All cells were measured between 25 – 28 °C. C-rates were calculated with respect to the theoretical discharge capacity for one electron reduction of heat-treated Pyr-IHF ($FeF_2OH \cdot 0.39\ H_2O$, 227 mA h g^{-1}).

To replace spent Li metal anodes with fresh ones, the cycled cells were transferred into a glove box (GB) and opened with a decrimping machine. Then, the spent Li metal anode was replaced with a fresh one, and the coin cell was closed with a crimping machine.

SUPPLEMENTARY TEXT

Operando Heat Treatment of Pyr-IHF

To determine the optimal temperature for removing templating H_2O molecules from within the interconnected channels in as-synthesized Pyr-IHF, and thereby improve Li-ion conductivity, as-synthesized Pyr-IHF was characterized during heating to 350 °C using *in-situ* synchrotron X-ray diffraction (**Figure 1**d, Figure S5). The minor $FeF_3(H_2O)_2 \cdot H_2O$ impurities amorphized upon heating to 120 °C, while the H_2O content of Pyr-IHF gradually decreased until 240 °C, when all H_2O had been extracted from the channels (Figure S6, Table S5). Subsequently, the Pyr-IHF phase rapidly vanished, indicating that H_2O loss is destabilizing the porous pyrochlore structure, leading to its collapse.[17]

Analysis of the pattern at 290 C indicates the β-Fe_2O_3 with the cubic bixbyite structure ($Ia\bar{3}$) and a lattice constant of 9.55 Å, which is significantly larger than the 9.40 Å reported for pure Fe_2O_3 (Figure S7). It therefore seems likely that the β-Fe_2O_3 phase exhibits significant F^- substitution, given the smaller lattice energy from partial F^- substitution, and accordingly larger lattice constant. This is consistent with the fact that the iron hydroxy fluoride precursor also has mixed OH/F substitution in Pyr-IHF.[1]

Interestingly, the obtained β-Fe_2O_3 is a metastable polymorph of Fe_2O_3, instead of the thermodynamically stable α-Fe_2O_3 with a corundum structure. We speculate that the β-Fe_2O_3 phase

forms because it is structurally related to Pyr-IHF, and can therefore be obtained *via* a topotactic process. In Pyr-IHF, four corner-sharing $Fe(F/OH)_6$ octahedra form a tetrahedron with an octahedral void. These tetrahedra of $Fe(F/OH)_6$ octahedra are then interconnected with the other tetrahedral units to form chains along the 110 and equivalent directions through corner-sharing of the $Fe(F/OH)_6$ octahedra. β-Fe_2O_3 has the same structural subunit of (distorted) $Fe(F/OH)_6$ octahedra that form tetrahedra with an octahedral void. However, these tetrahedra are connected through edge-sharing. Hence, every second tetrahedral unit will rotate slightly to form mutually edge-sharing $Fe(F/OH)_6$ octahedra, thereby collapsing the octahedral void that was previously present. Moreover, the second 16 c site in Pyr-IHF (0.5 0 0) is unoccupied, resulting in the presence of the observed channels. However, a collapse of the channels, and a corresponding occupation of the 16 c site with Fe can recover the bixbyite structure. Subsequently, the anionic ligands that are released through the edge-sharing can fill the anion sublattice of the open-packed structure in Pyrochlore to complete the distorted bcc anion sublattice of the bixbyite structure. The formation of β-Fe_2O_3 is therefore accessible through a topotactic transformation, and thereby kinetically more accessible than the thermodynamically stable α-Fe_2O_3.

MCR-ALS Analysis

By decomposing the obtained spectra into its individual components, for which the oxidation states are well-known, linear combination of the components yields the average Fe oxidation state during any point of charge, and therefore the Fe-based capacity contribution. Initial PCA indicated the presence of two components in the spectrum (Figure S15). In principle, MCR analysis can be constrained by any number of components, but to not overfit the data, a two component system was chosen in accordance with PCA analysis to constrain the MCR analysis. As the initial guesses for the two components, the XANES spectra for heat-treated Pyr-IHF (Fe^{+III}) and FeF_2 (Fe^{+II}) were used and then subsequently refined (Figure S16).

The initial mean oxidation state is 2.91 (91% Fe^{+III}/ 9% Fe^{+II}). Overall, the charge for Fe reduction/ oxidation follows the electrochemically determined charge very closely up to full discharge, indicating that no significant side reactions contribute to the capacity. The final mean oxidation state is 2.11 (11% Fe^{+III}/ 89% Fe^{+II}), corresponding to a capacity of 181 mA h g^{-1},

The observed small deviations of the charge for Fe reduction/ oxidation relative to the electrochemically determined charge are expected from slightly inhomogeneously distributed active material inside the cathode, causing the fraction of the active material illuminated by the X-ray beam (*ca.* 2.5% of the total electrode volume) to be reduced at slightly different potentials than expected from the voltage profile.

Ex-situ PDF Analysis

In the sample discharged to 2.5 V, atom pair correlations are detected until *ca*. 9 Å (Figure 3e). In the low *r*-range, the first-coordination Fe-F/OH bond peak shifts to higher values (2.05 Å *vs.* 1.95 Å at 3 V), consistent with a reduction of Fe^{+III} to Fe^{+II}. Moreover, the peak broadens significantly, suggesting a wider distribution of bond distances and likely strongly distorted $Fe(F/OH)_6$ octahedral environments (Figure S21, S26). Concomitantly, three distinguishable peaks appear between 3 – 4 Å, indicating multiple second-shell Fe-Fe correlations and correspondingly different $Fe(F/OH)_6$ octahedral connectivities: The first peak at 3.30 Å corresponds to edge-sharing $Fe(F/OH)_6$ octahedra, similar to the Fe-Fe distance in other $FeF_{2-x}(OH)_x$ phases with edge-sharing octahedra, e.g. 3.30°Å in rutile FeF_2,[18] and 3.27°Å in diaspore-type Fe(OH)F.[19] Given the Fe-F/OH bond distance of 2.05 Å, a Fe-F/OH-Fe bond angle of 107° is obtained, suggesting a significant distortion from the 90° angle of an ideal octahedral environment (Figure S26b). This is commonly observed in edge-sharing octahedra as a result of coulombic repulsion between the metal ions. For instance, rutile FeF_2 also exhibits strongly distorted octahedral environments with a Fe-F-Fe of 103°.[18]

Meanwhile the peaks at 3.60 Å and the shoulder at 3.80 Å indicate corner-sharing $Fe(F/OH)_6$ octahedra with Fe-F/OH-Fe bond angles of 123° and 136°, respectively (Figure S26b). While a bond angle of 136° is consistent with the Fe-F/OH-Fe bond angles observed in Pyr-IHF (139°-142°), the reduced Fe-F/OH-Fe bond angle of 123° is not observed in unsubstituted iron fluorides, because of the strong coulombic repulsion between anionic ligands of neighboring FeF_6 octahedra. For instance, the Fe-F-Fe bond angles are significantly higher with 142°, 145° and 158° in HTB-FeF_3,[20] and 152° in r-FeF_3.[21] However, smaller bond angles may be observed in the presence of hydroxide ligands, where hydrogen bridges between nearby anionic ligands may shield their

anionic charge, and stabilize anionic ligands in close proximity to each other. For instance iron (III) oxy hydroxides,[22] and iron (II) hydroxy fluorides,[19] can both exhibit Fe-X-Fe bond angles around 120°, often accompanied by a rotation around the Fe-X-Fe-X dihedral angle. We note that Li^+ may shield adjacent anionic ligand charge similar to H^+, so the presence of protons may not necessarily be required for the existence of such strongly reduced Fe-F/OH-Fe bond angles.

Iron (II) hydroxy fluoride (space group *Pnma*) is composed of edge-, and corner-sharing $Fe(F/OH)_6$ octahedra with similar bond distances and angles like the ones encountered for the discharged cathode (Figure S26c).[19] Moreover, the structure exhibits hexagonal channels, similar to Pyr-IHF, although they are highly distorted compared to those of Pyr-IHF. A refinement of the low-*r*-region (1.7 to 7 Å) of the Pyr-IHF cathode discharged to 2.5 V yielded satisfactory agreement with the structure of iron (II) hydroxy fluoride (R_W = 20.7%), making the hexagonal channels a plausible structural motif in amorphized discharged Pyr-IHF cathodes.

While the multiple peaks observed in the low-*r*-region can be fitted with a single iron (II) hydroxy fluoride phase containing hexagonal channels, the PDF data would also be consistent with the presence of multiple amorphous phases. Regardless of the precise local structure however, the PDF data is incommensurate with a more densely packed anion framework, like the one encountered in r-FeF_3.(*ca.* 30 vol-% denser than Pyr-IHF). Instead, the electrochemically active amorphous phase appears to have a density comparable to Pyr-IHF, and may therefore retain a sufficiently high fraction of interconnected channels to maintain a Li-ion percolating network, as well as the ability to store Li-ions within the channels. The similar density of the amorphous phase relative to the Pyrochlore phase is further supported by the negligible volume changes of the 250 nm large particles during lithiation (Figure 2d-h).

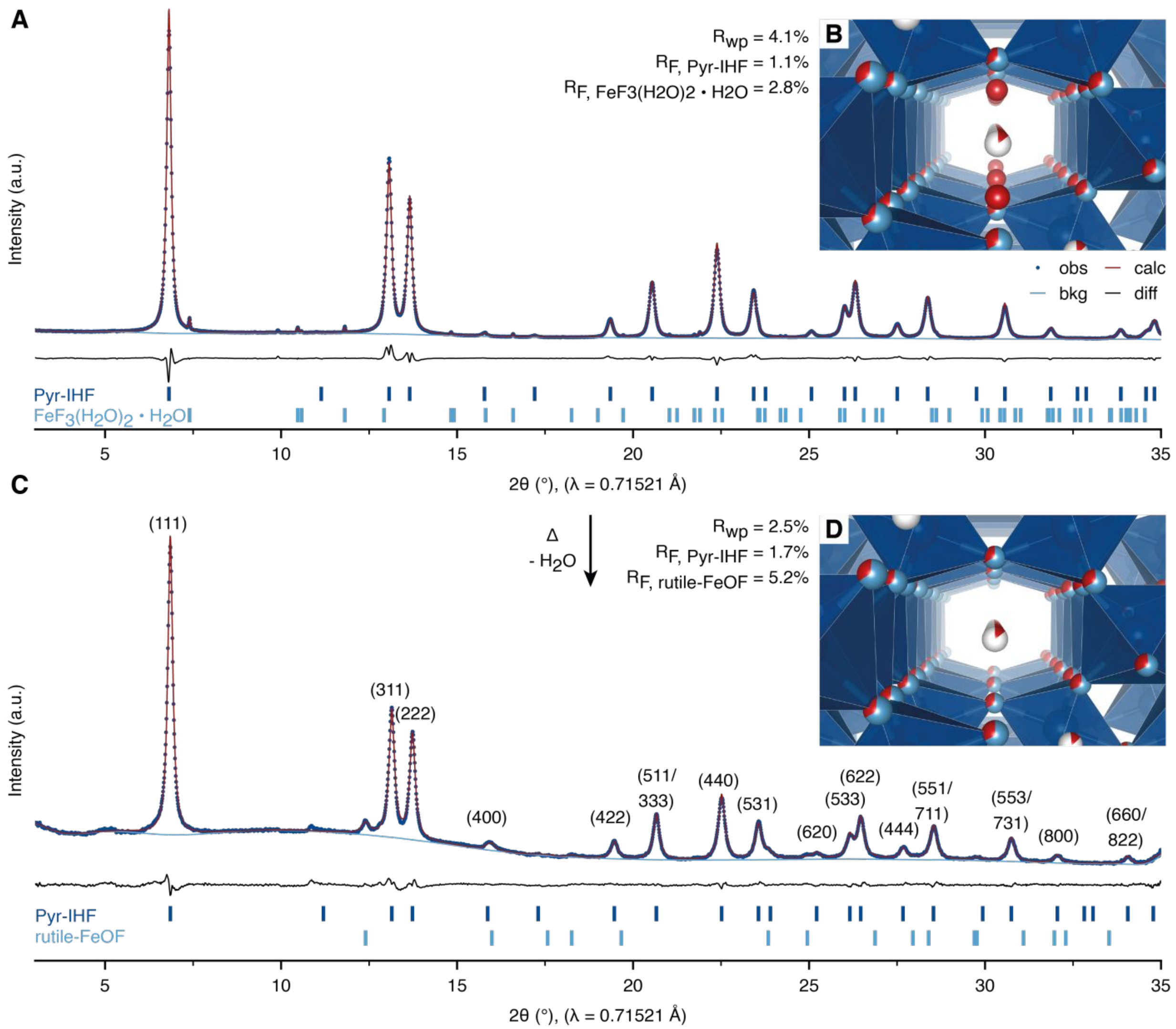

Figure S1. Rietveld refinement of the synchrotron powder diffraction patterns of as-synthesized Pyr-IHF (**A**) and Pyr-IHF heat-treated to 275 °C (**C**). Crystal structure of the crystal water within the channels for as-synthesized Pyr-IHF (**B**) and heat-treated Pyr-IHF (**D**).

Table S1. Overview of the Rietveld-refined parameters for as-synthesized Pyr-IHF. Values in brackets indicate estimated standard error. If no error is given, the parameter was not refined.

S.G $Fd\bar{3}m$[1]		ρ = 2.867 g cm^{-3}		x = 98.39(7) wt.-%		
a = b = c = 10.42162(14) Å			α = β = γ = 90°			
Site	Wyck.	x	y	y	Occ.	U_{iso} (Å^2)
Fe1	16c	0	0	0	1	0.0048(3)
F1	48f	0.31577(13)	⅛	⅛	1	0.0161(6)
O1	8b	⅜	⅜	⅜	1	0.1
O2	16d	¾	¾	½	0.081(5)	0.1
No. reflections = 29		No. observations = 3017		No. parameters = 34		
χ^2 = 1169		χ^2_{red} = 0.39		R_w = 4.1%		R_F = 1.1%

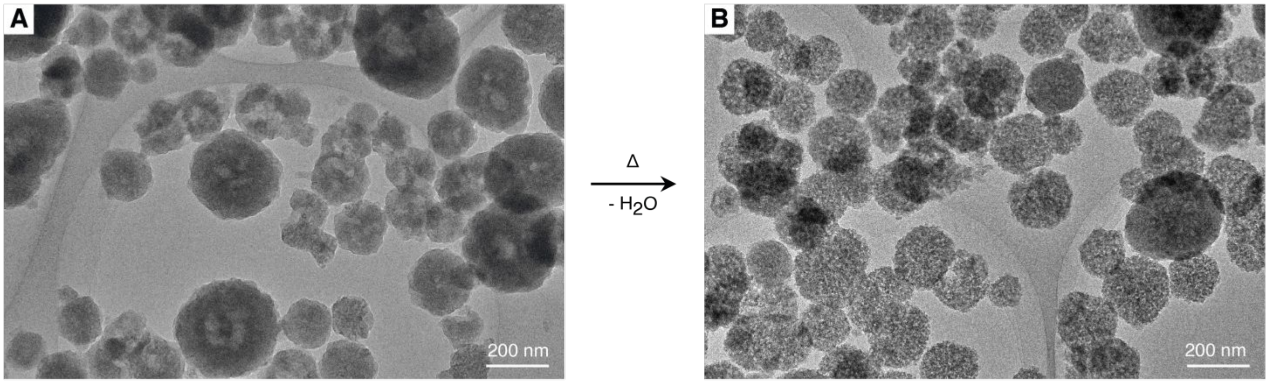

Figure S2. TEM micrographs of as-synthesized Pyr-IHF (**A**) and heat-treated Pyr-IHF (**B**).

Table S2. Overview of the Rietveld-refined parameters for heat-treated Pyr-IHF. Values in brackets indicate estimated standard error. If no error is given, the parameter was not refined.

S.G $Fd\bar{3}m$[1]		ρ = 2.865 g cm^{-3}		x = 92.6(3) wt.-%		
a = b = c = 10.36219(23) Å			$\alpha = \beta = \gamma = 90°$			
Site	Wyck.	x	y	y	Occ.	U_{iso} (Å^2)
Fe1	16c	0	0	0	1	0.0090(4)
F1	48f	0.31147(18)	⅛	⅛	1	0.0095(6)
O1	8b	⅜	⅜	⅜	0.806(9)	0.1
O2	16d	¾	¾	½	0.043(6)	0.1
No. reflections = 28		No. observations = 3017		No. parameters = 34		
χ^2 = 259		χ^2_{red} = 0.09		R_w = 2.5%		R_F = 1.7%

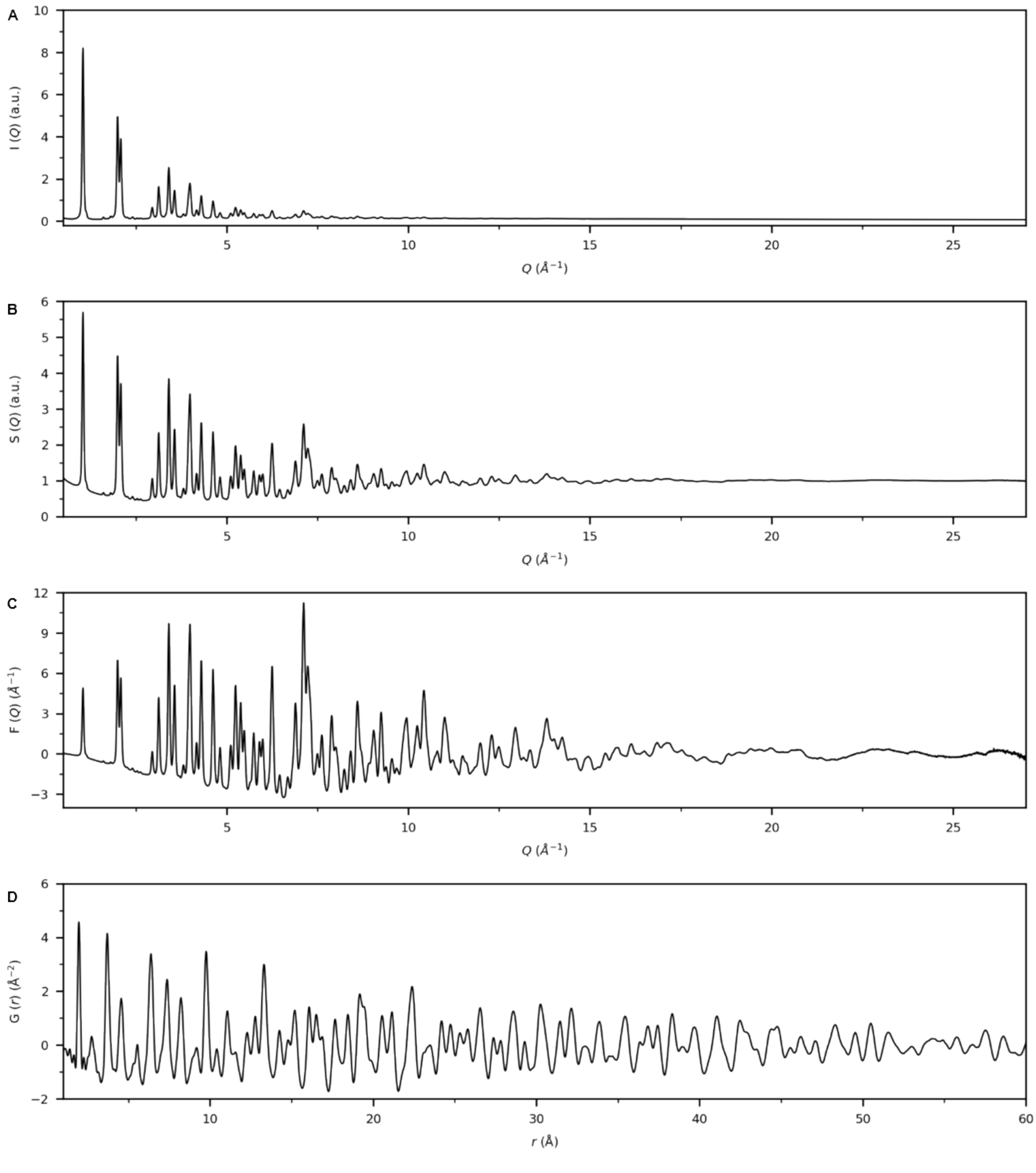

Figure S3. Synchrotron X-ray total scattering of as-synthesized Pyr-IHF.

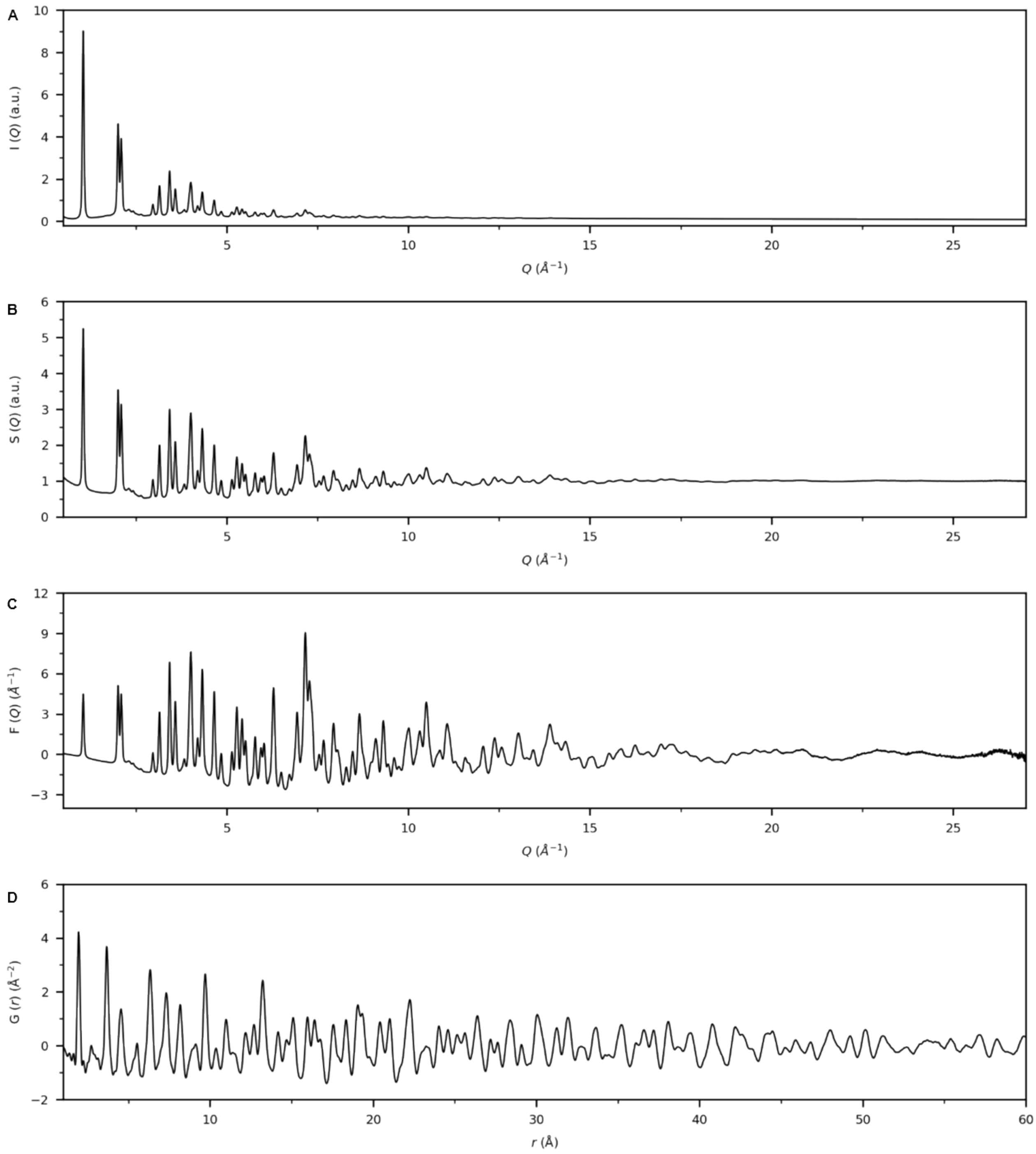

Figure S4. Synchrotron X-ray total scattering of heat-treated Pyr-IHF.

Table S3. Overview of the refined parameters from PDF for as-synthesized Pyr-IHF. Values in brackets indicate estimated standard error. If no error is given, the parameter was not refined.

S.G $Fd\bar{3}m$[1]				x = 100 wt.-%		
a = b = c = 10.4307(67) Å				α = β = γ = 90°		
Site	Wyck.	x	y	y	Occ.	U (Å^2)
Fe1	16c	0	0	0	1	U_{11} = 0.0055(14), U_{12} = -0.0012(13)
F1	48f	0.3144(21)	⅛	⅛	1	U_{11} = 0.017(9) , U_{22} = 0.021(7) , U_{23} = 0.010(9)
O1	8b	⅜	⅜	⅜	0.57(45)	U_{iso} = 0.1
O2	16d	¾	¾	½	0.35(30)	U_{iso} = 0.1
No. parameters = 13			χ^2 = 8.4		χ^2_{red} = 0.023	R_w = 13.0%

Table S4. Overview of the refined parameters from PDF for heat-treated Pyr-IHF. Values in brackets indicate estimated standard error. If no error is given, the parameter was not refined.

S.G $Fd\bar{3}m$[1]				x = 90(3) wt.-%				
a = b = c = 10.3662(38) Å				α = β = γ = 90°				
Site	Wyck.	x	y	y	Occ.	U (Å^2)		
Fe1	16c	0	0	0	1	U_{11} = 0.0055(4), U_{12} = -0.0010(7)		
F1	48f	0.3127(12)	⅛	⅛	1	U_{11} = 0.017(5) , U_{22} = 0.020(4) , U_{23} = 0.007(4)		
O1	8b	⅜	⅜	⅜	0.26(27)	U_{iso} = 0.1		
O2	16d	¾	¾	½	0.49(19)	U_{iso} = 0.1		
No. parameters = 16			χ^2 = 32			χ^2_{red} = 0.017		R_w = 13.8%

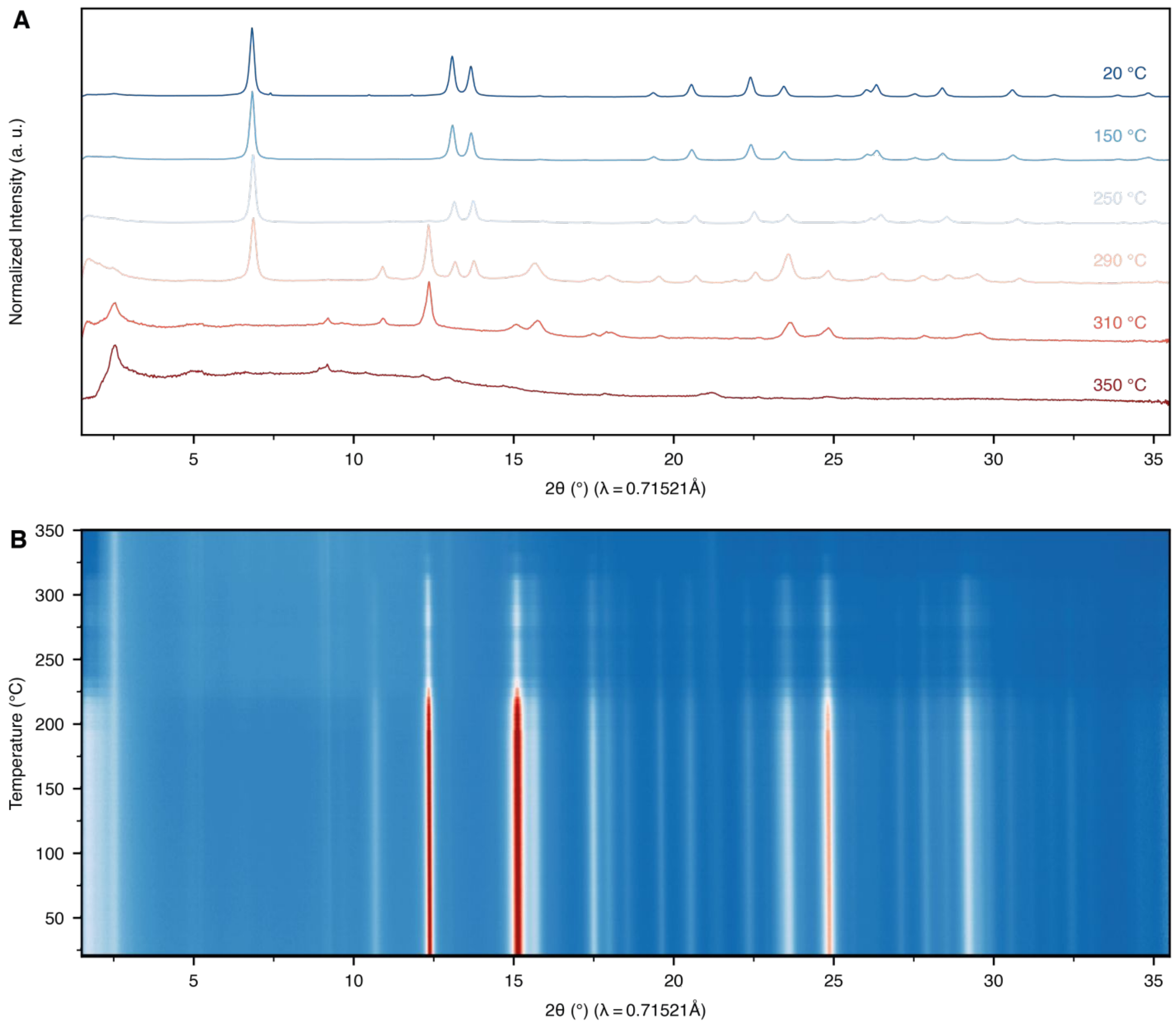

Figure S5. *In-situ* synchrotron XRD during heat treatment of as-synthesized Pyr-IHF. **A**) shows selected XRDs during heating and **B**) shows the evolution during cooldown.

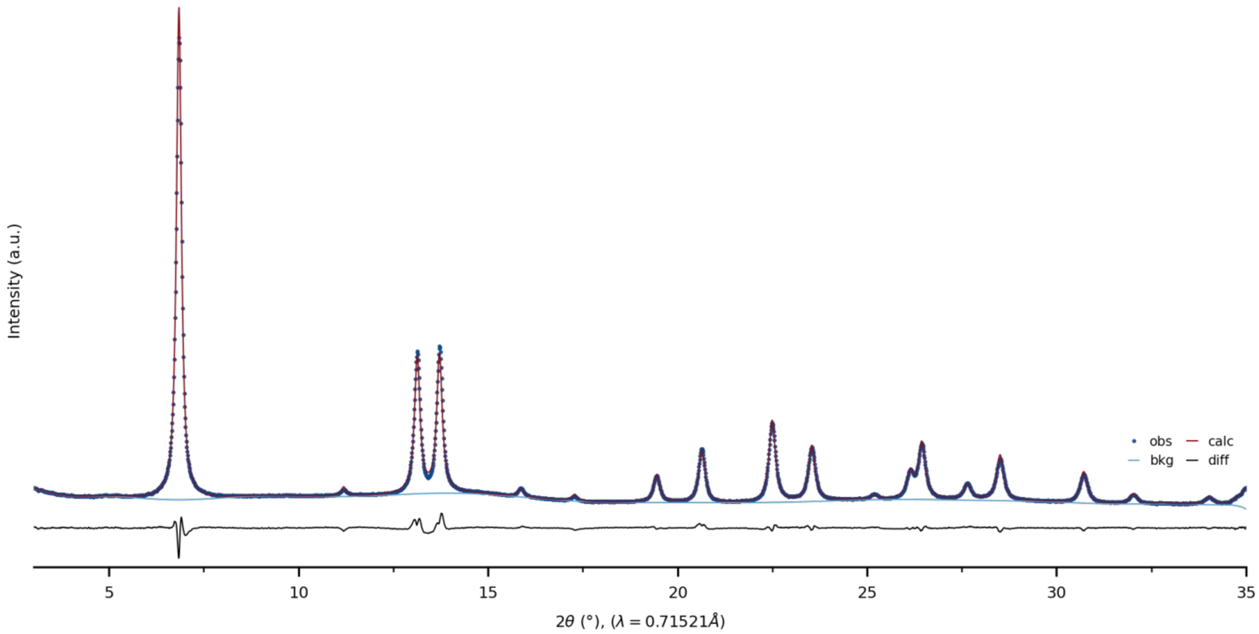

Figure S6. Rietveld refinement of the synchrotron powder diffraction patterns of Pyr-IHF at 250 °C. The refined values are summarized in Table S5.

Table S5. Overview of the Rietveld-refined parameters for heat-treated Pyr-IHF at 250 °C. Values in brackets indicate estimated standard error. If no error is given, the parameter was not refined.

S.G $Fd\bar{3}m$[1]		ρ = 2.688 g cm^{-3}		x = 100 wt.-%		
a = b = c = 10.37067(25)Å			α = β = γ = 90°			
Site	Wyck.	x	y	y	Occ.	U_{iso} (Å^2)
Fe1	16c	0	0	0	1	0.0043(4)
F1	48f	0.31395(15)	⅛	⅛	1	0.0224(7)
No. reflections = 29		No. observations = 3017		No. parameters = 34		
χ^2 = 804		χ^2_{red} = 0.27		R_w = 3.7%		R_F = 1.6%

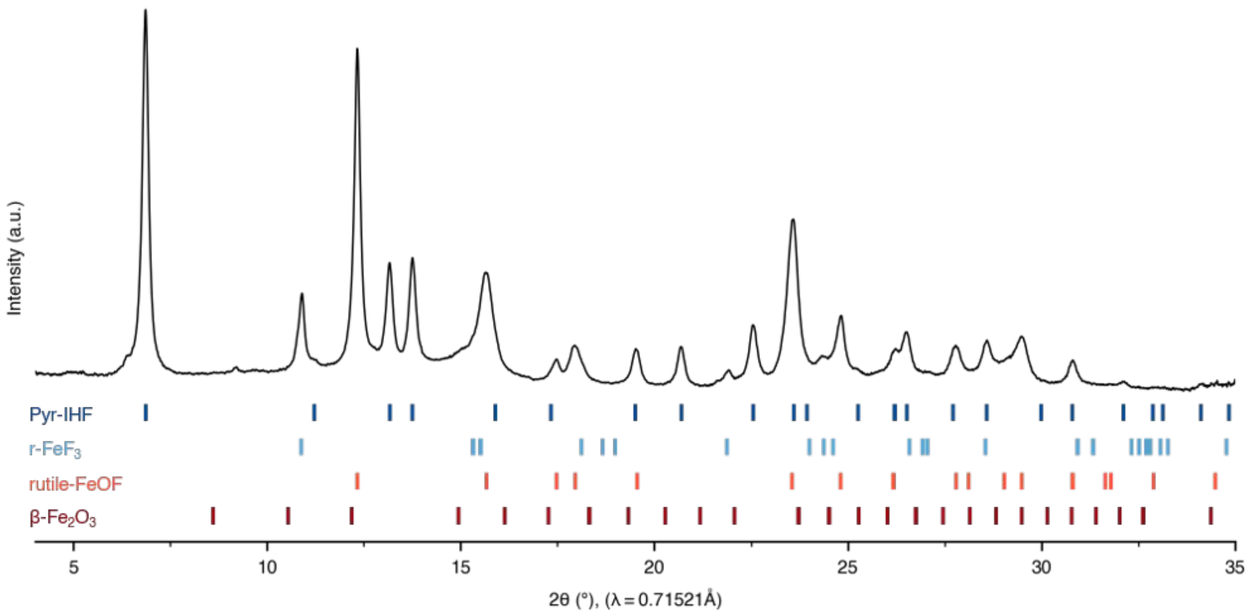

Figure S7. Synchrotron powder diffraction patterns of Pyr-IHF at 290 °C. During collapse of the pyrochlore structure, r-FeF_3, rutile-FeOF and β-Fe_2O_3 form as decomposition products.

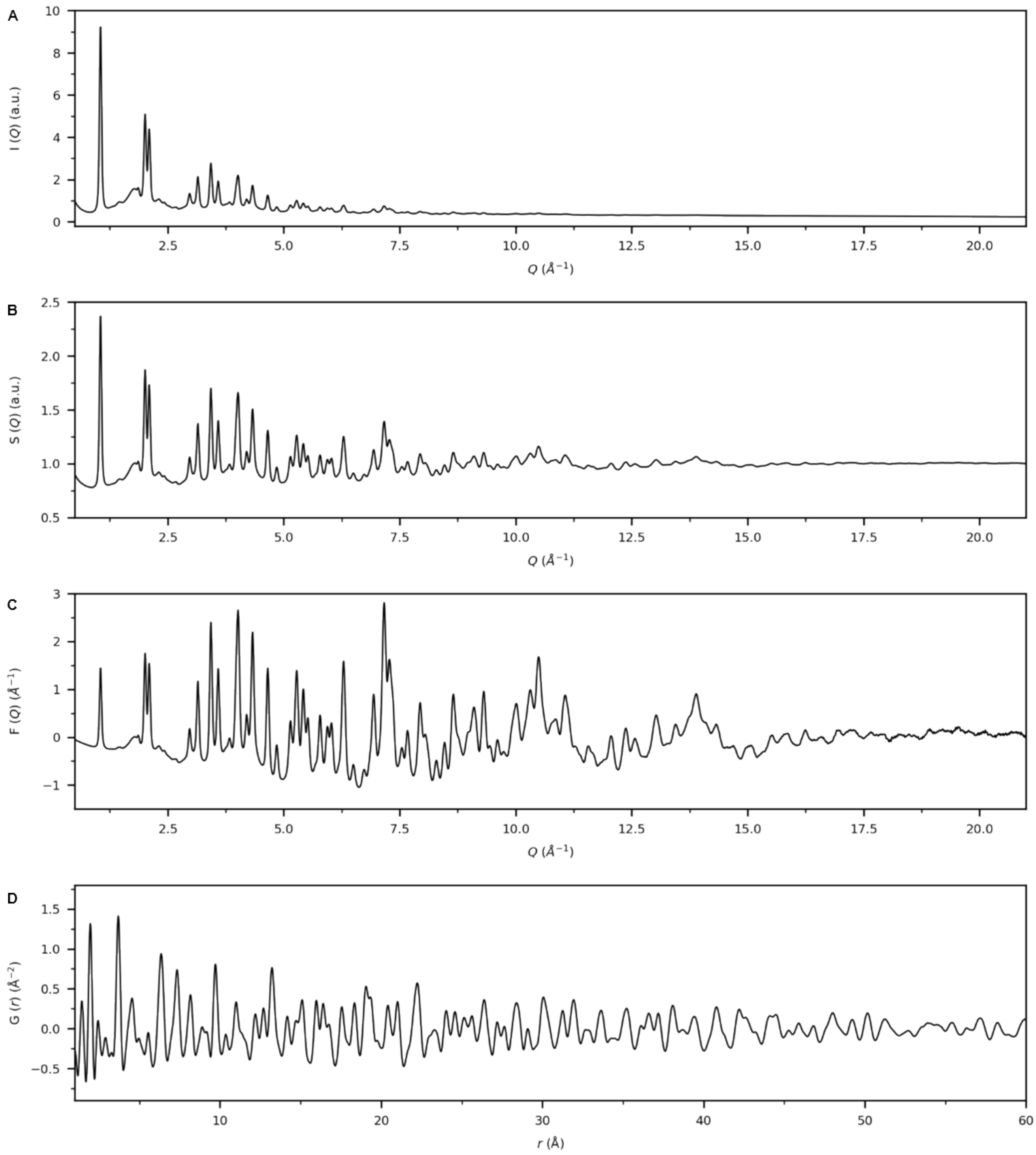

Figure S8. Synchrotron X-ray total scattering of pristine cathodes prepared in air containing Pyr-IHF heat-treated to 275°C prior to cathode preparation. The lack of any crystalline decomposition products observed in (A) highlights the stability of Pyr-IHF cathodes under air.

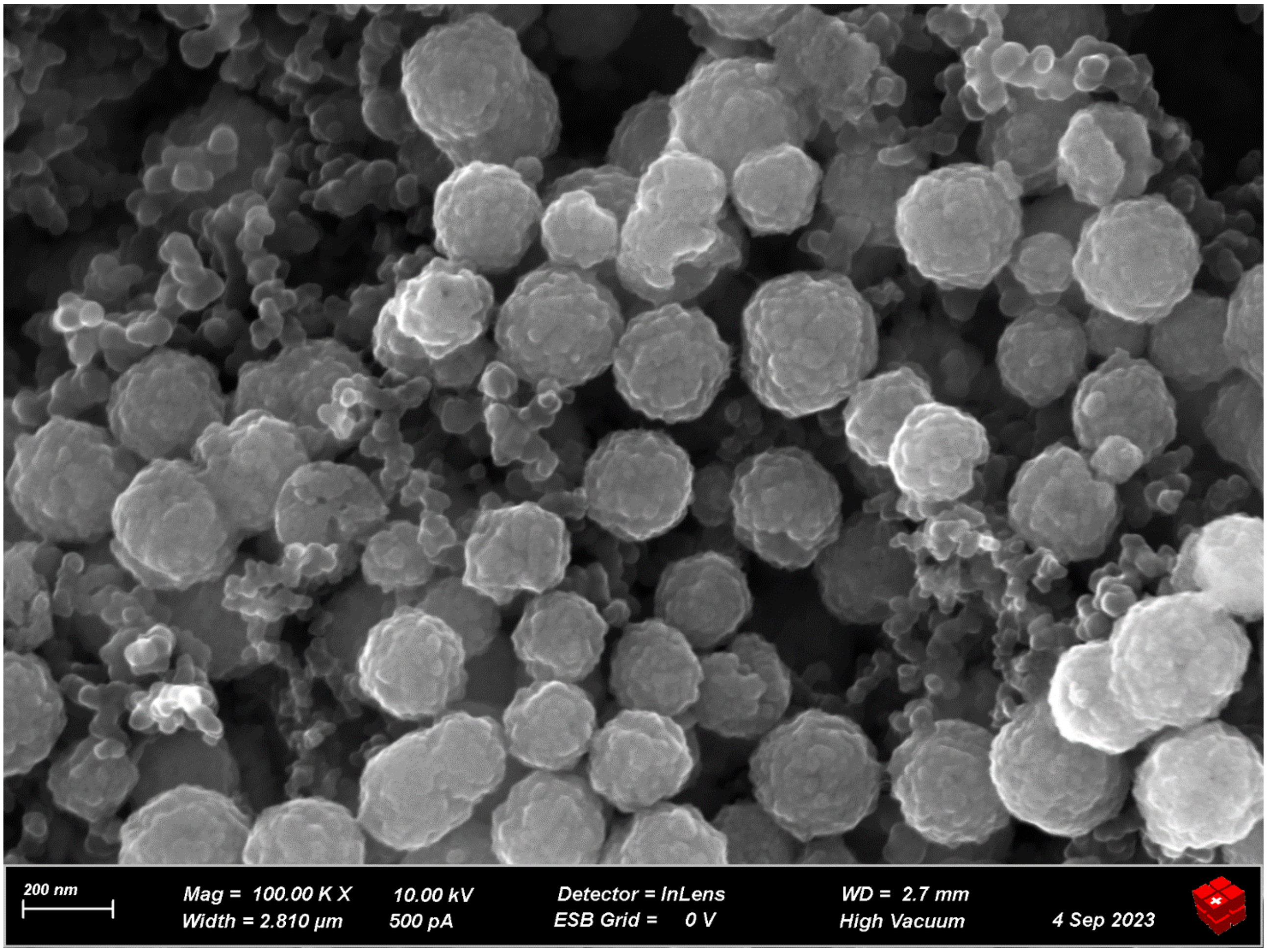

Figure S9. Uncolored SEM micrographs of pristine Pyr-IHF cathodes.

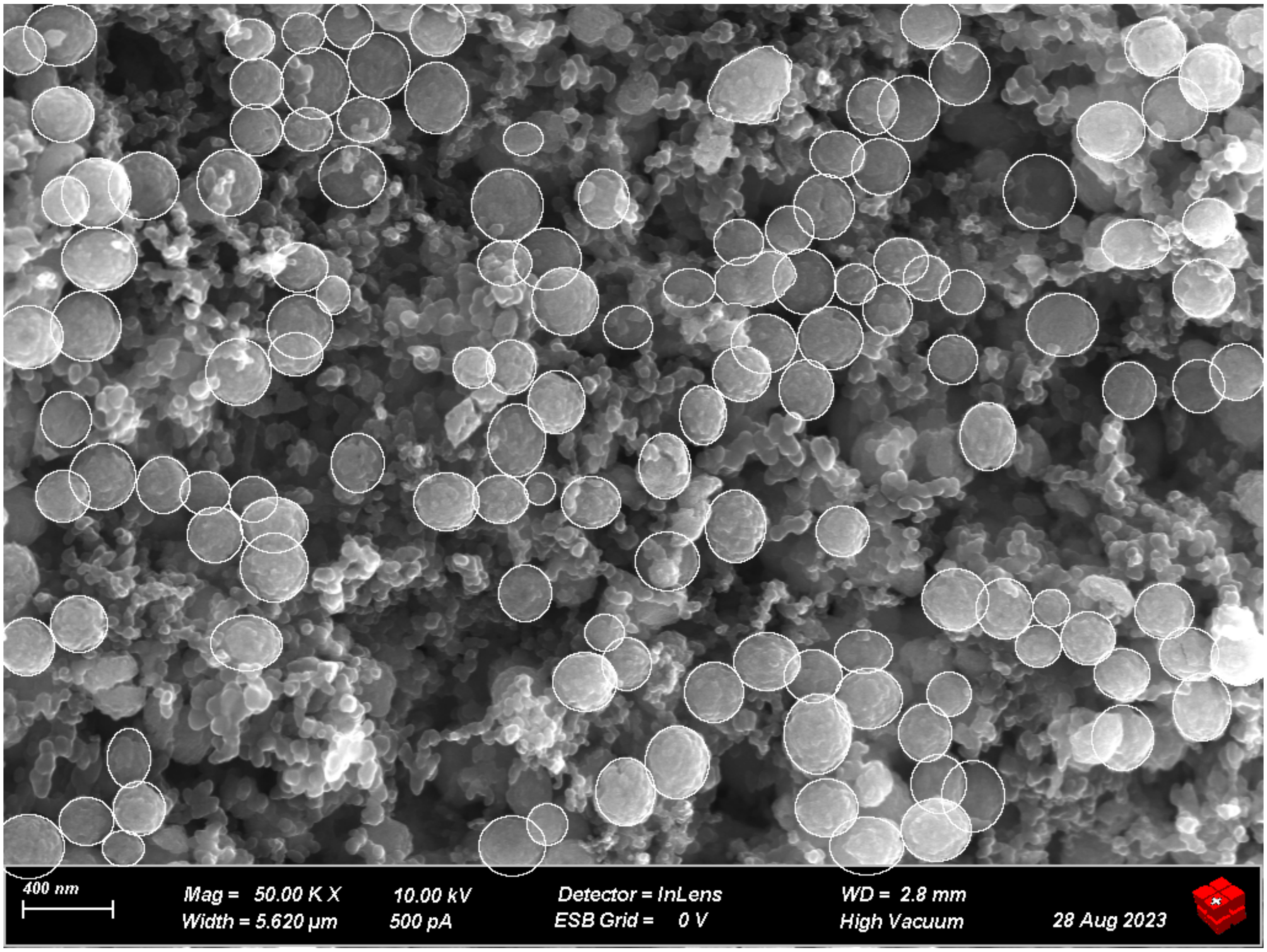

Figure S10. SEM micrographs of pristine Pyr-IHF cathodes used for the determination of the particle size distribution.

Table S6. Overview of particle size distributions used to evaluate the best fitting particle size distribution of Pyr-IHF cathodes before and after cycling.

Weibull 2P	$f(t) = \left(\frac{\beta}{\alpha}\right)\left(\frac{t}{\alpha}\right)^{\beta-1} e^{-\left(\frac{t}{\alpha}\right)^{\beta}}$	**(1)**
Weibull 3P	$f(t) = \left(\frac{\beta}{\alpha}\right)\left(\frac{t-\gamma}{\alpha}\right)^{\beta-1} e^{-\left(\frac{t-\gamma}{\alpha}\right)^{\beta}}$	**(2)**
Normal 2P	$f(t) = \frac{1}{\sigma\sqrt{2\pi}} \exp\left[-\frac{1}{2}\left(\frac{t-\mu}{\sigma}\right)^{2}\right]$	**(3)**
Gumbel 2P	$f(t) = \frac{1}{\sigma} e^{z-e^{z}}$ with $z = \frac{t-\mu}{\sigma}$	**(4)**
Gamma 2P	$f(t) = \frac{t^{\beta-1}}{\Gamma(\beta)\alpha^{\beta}} e^{-\frac{t}{\alpha}}$ with $\Gamma(\beta) = \int_{0}^{\infty} t^{\beta-1} e^{-t} \mathrm{d}t$	**(5)**
Gamma 3P	$f(t) = \frac{\gamma t^{\beta-1}}{\Gamma\left(\frac{\beta}{\gamma}\right)\alpha^{\beta}} e^{-\left(\frac{t}{\alpha}\right)^{\gamma}}$ with $\Gamma\left(\frac{\beta}{\gamma}\right) = \int_{0}^{\infty} t^{\frac{\beta}{\gamma}-1} e^{-t} \mathrm{d}t$	**(6)**
Lognormal 2P	$f(t) = \frac{1}{\sigma t\sqrt{2\pi}} \exp\left[-\frac{1}{2}\left(\frac{\ln(t)-\mu}{\sigma}\right)^{2}\right]$	**(7)**
Lognormal 3P	$f(t) = \frac{1}{\sigma(t-\gamma)\sqrt{2\pi}} \exp\left[-\frac{1}{2}\left(\frac{\ln(t-\gamma)-\mu}{\sigma}\right)^{2}\right]$	**(8)**
Loglogistic 2P	$f(t) = \frac{\left(\frac{\beta}{\alpha}\right)\left(\frac{t}{\alpha}\right)^{\beta-1}}{\left(1+\left(\frac{t}{\alpha}\right)^{\beta}\right)^{2}}$	**(9)**
Loglogistic 3P	$f(t) = \frac{\left(\frac{\beta}{\alpha}\right)\left(\frac{t-\gamma}{\alpha}\right)^{\beta-1}}{\left(1+\left(\frac{t-\gamma}{\alpha}\right)^{\beta}\right)^{2}}$	**(10)**
Exponential 1P	$f(t) = \lambda e^{-\lambda t}$	**(11)**
Exponential 2P	$f(t) = \lambda e^{-\lambda(t-\gamma)}$	**(12)**

Table S7. Fitted probability densities for the particle size distribution of the pristine Pyr-IHF cathodes before cycling (N = 133). α, β, γ, μ, σ and λ are the parameters used to fit the probability distributions. The Bayesian Information Criterion (BIC) was used to determine the goodness of the fit.

Distribution	α	β	γ	μ	σ	λ	BIC
Weibull 2P	262	7.31					1359
Normal 2P				245	39.1		1362
Weibull 3P	200	5.51	60.5				1363
Gumbel 2P				264	35.0		1365
Gamma 2P	6.67	36.7					1369
Gamma 3P	6.67	36.7	0				1374
Lognormal 2P				5.49	0.169		1374
Loglogistic 2P	245	10.5					1374
Lognormal 3P			0	5.49	0.169		1379
Loglogistic 3P	245	10.5	0				1379
Exponential 2P			136			0.00920	1522
Exponential 1P						0.00447	1736

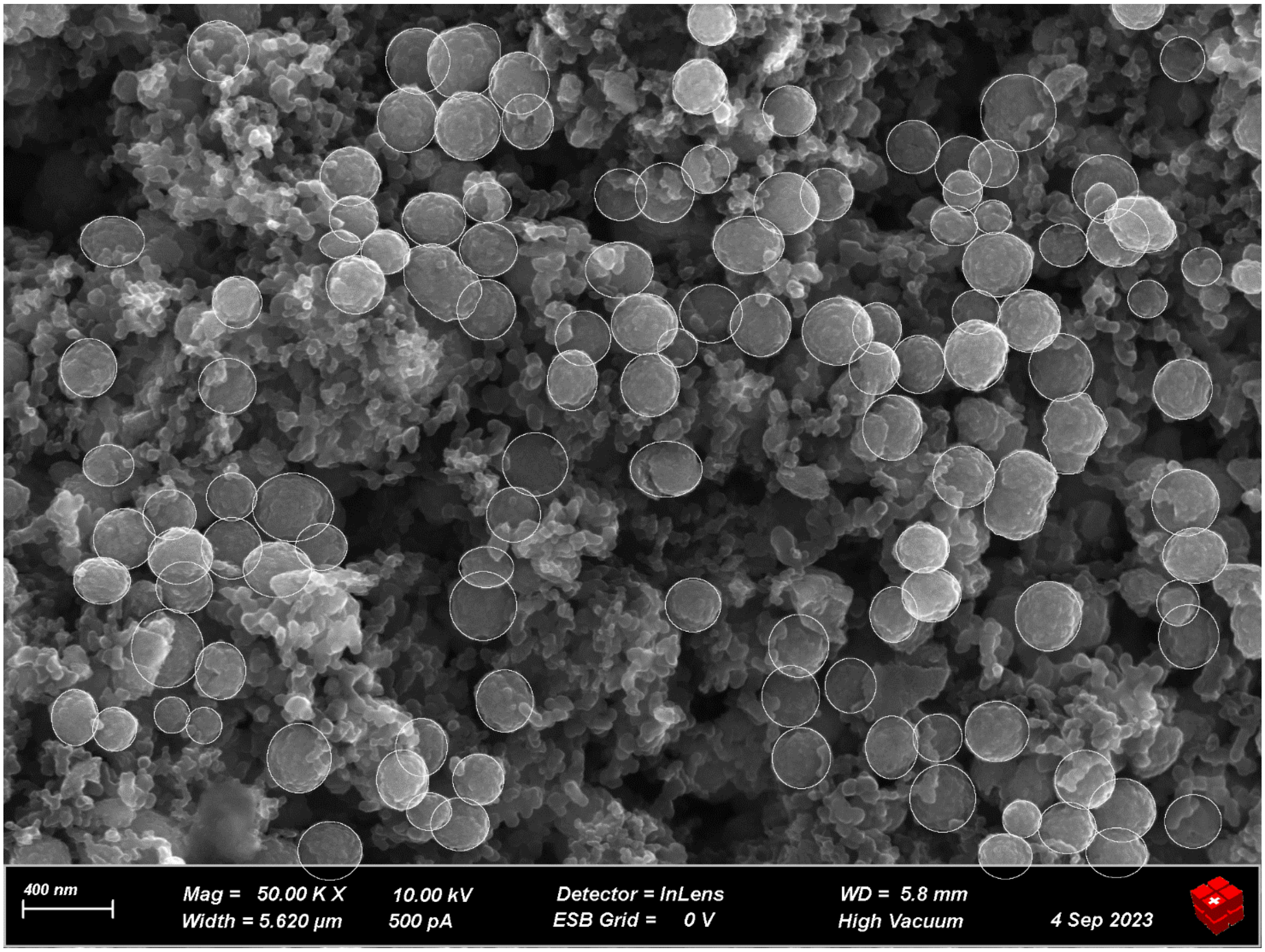

Figure S11. Uncolored SEM micrographs of Pyr-IHF cathodes discharged to 2 V, and used for the determination of the particle size distribution.

Table S8. Fitted probability densities for the particle size distribution of the Pyr-IHF cathodes discharged to 2 V (*N* = 122). α, β, γ, μ, σ and λ are the parameters used to fit the probability distributions. The Bayesian Information Criterion (BIC) was used to determine the goodness of the fit.

Distribution	α	β	γ	μ	σ	λ	BIC
Weibull 2P	259	6.63					1264
Normal 2P				242	41.4		1265
Weibull 3P	171	4.28	86.34				1267
Gamma 2P	7.57	32.0					1270
Gumbel 2P				262	38.8		1274
Loglogistic 2P	242	9.87					1274
Lognormal 2P				5.47	0.181		1274
Gamma 3P	7.57	32.0	0				1275
Loglogistic 3P	242	9.87	0				1279
Lognormal 3P			0	5.47	0.181		1279
Exponential 2P			147			0.01049	1366
Exponential 1P						0.00456	1589

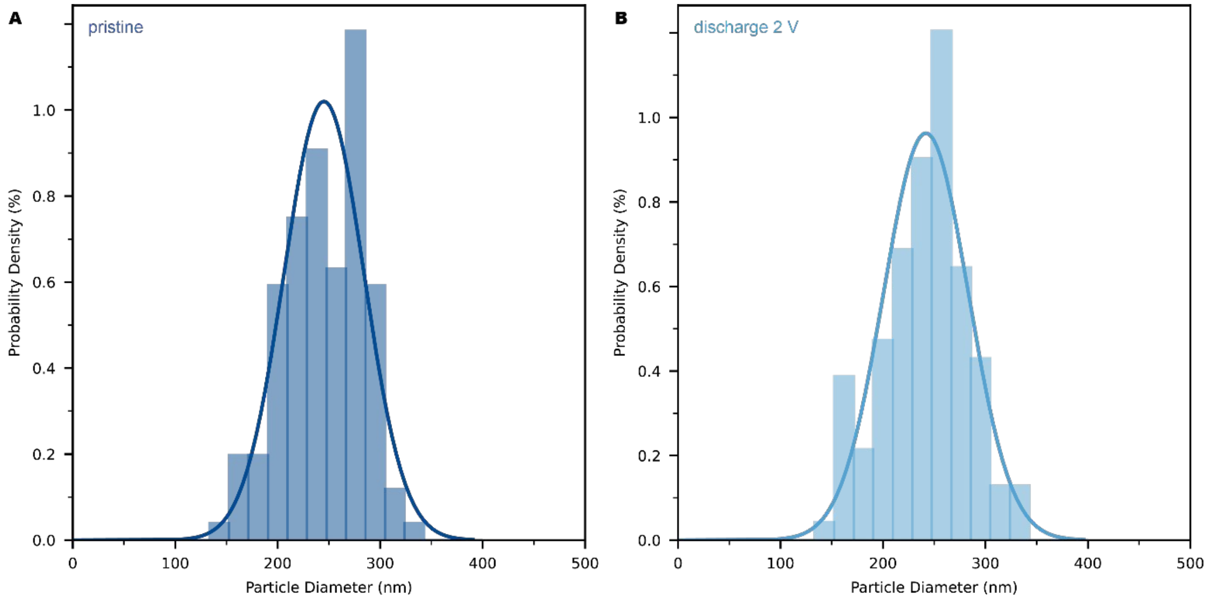

Figure S12. Particle size distribution of Pyr-IHF particles in cathodes after preparation (*N* = 133) (**A**) and after discharge to 2 V vs. Li+/Li (*N* = 122) (**B**). The histogram shows the measured particle size distribution, and the line indicates the fitted normal distribution.

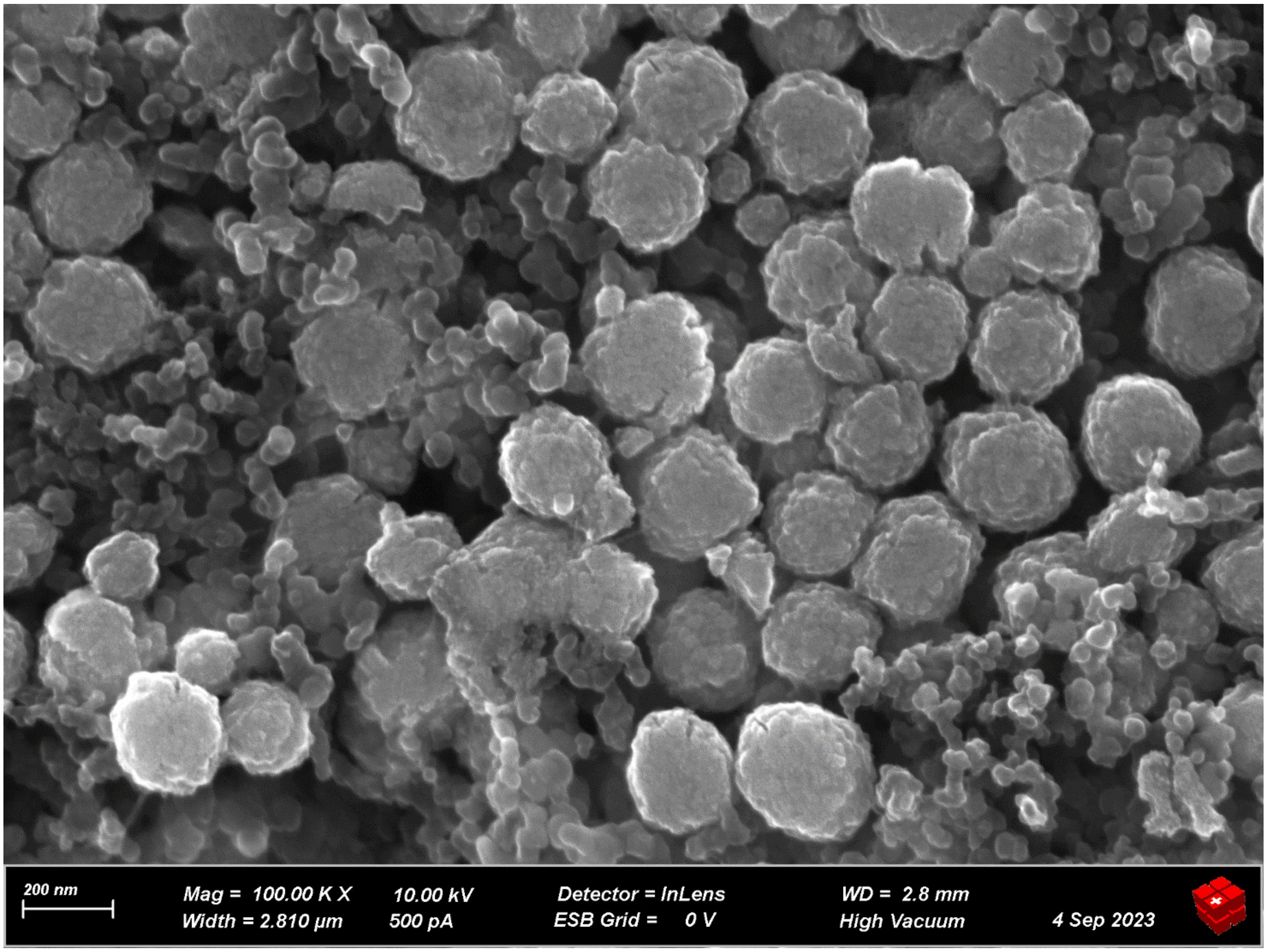

Figure S13. Uncolored SEM micrographs of Pyr-IHF cathodes cycled for 100 cycles (2 – 4.2 V vs. Li^+/Li), and used for the determination of the particle size distribution

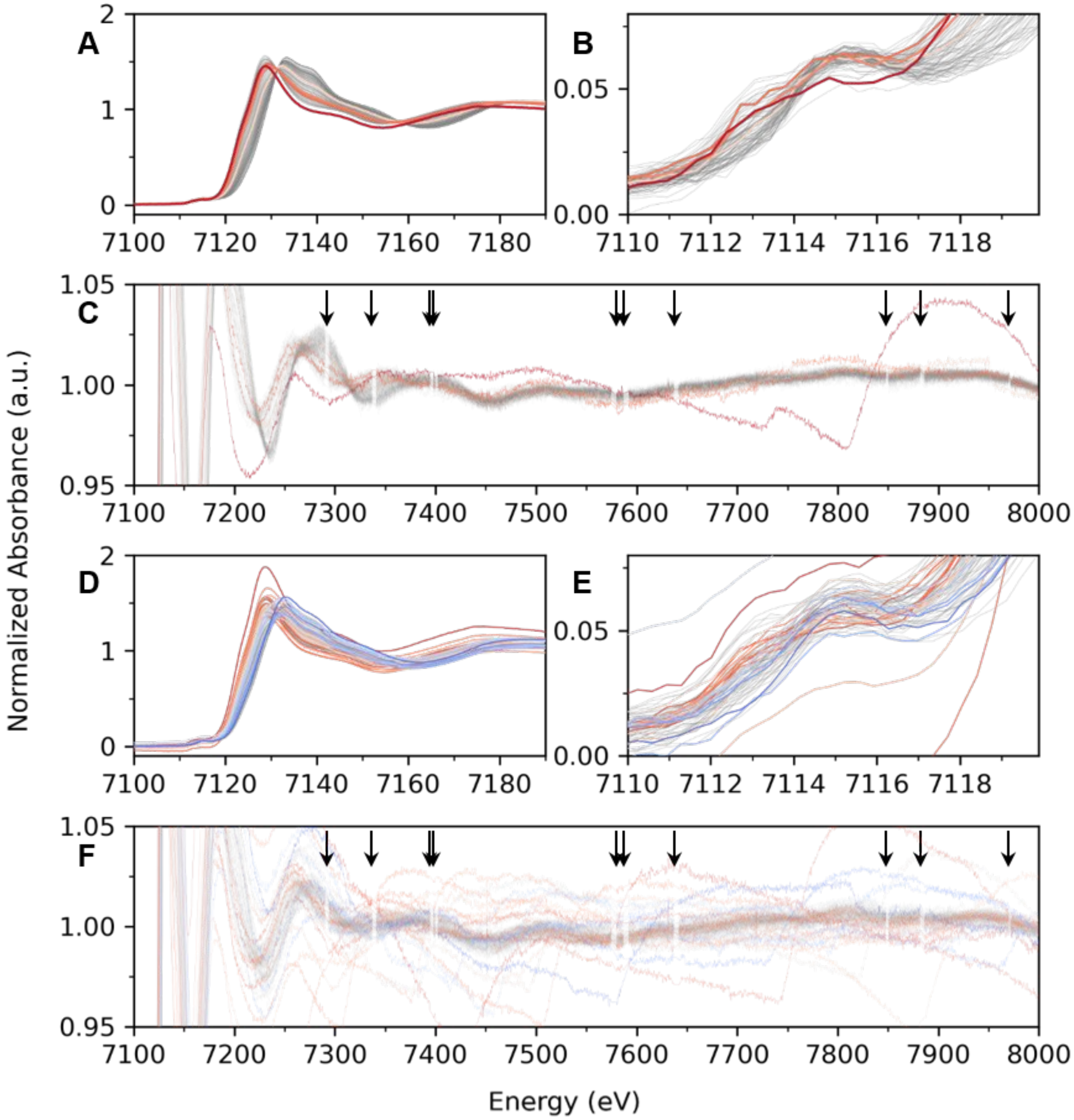

Figure S14. Normalized XAS spectra at the Fe *K*-edge before averaging during first discharge (**A-C**) and first charge (**D-F**) with magnification of the XANES-region (**A, D**), pre-edge feature (**B, E**), and EXAFS region (**C, F**). The colored spectra were excluded for analysis due to measurement errors, visible by strong deviations in the EXAFS region. The grey spectra were used for subsequent analysis. The arrows indicate regions were data was deglitched due to artifacts from the beamline optics.

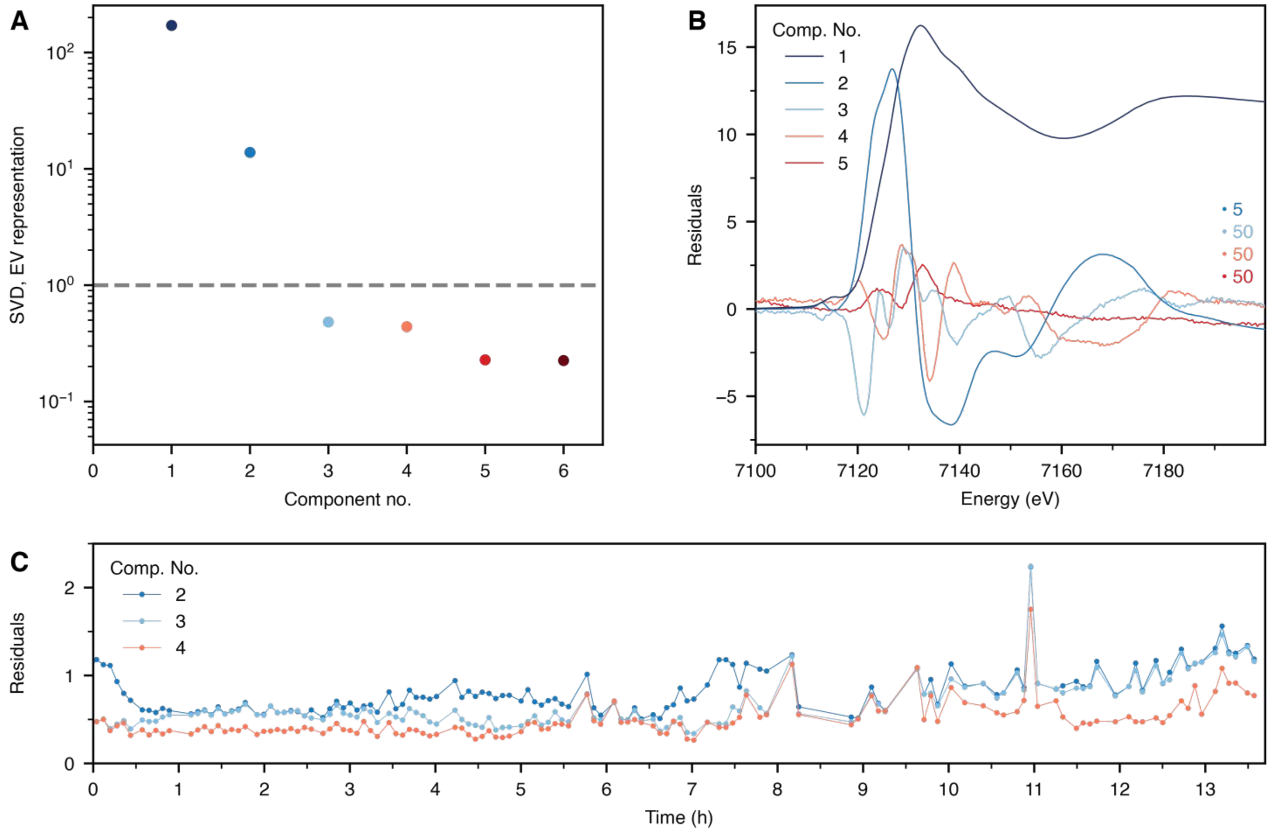

Figure S15. Principal component analysis for the *operando* XAS spectra full cells with Pyr-IHF containing cathodes. **A**) shows the singular value decomposition (SVD) in eigenvalue (EV) representation for the number of components. The dotted line indicates the cutoff value of 1 according to Kaiser's criterion.[12] **B**) shows the residuals of the individual components. The second to fifth component were scaled up by the respective factors on the right for improved readability. **C**) shows the residuals for each spectrum over the entire first cycle using two, three or four components. As the fit does not improve meaningfully when a third component is added to describe the spectra, only two components were used for subsequent MCR-ALS analysis.

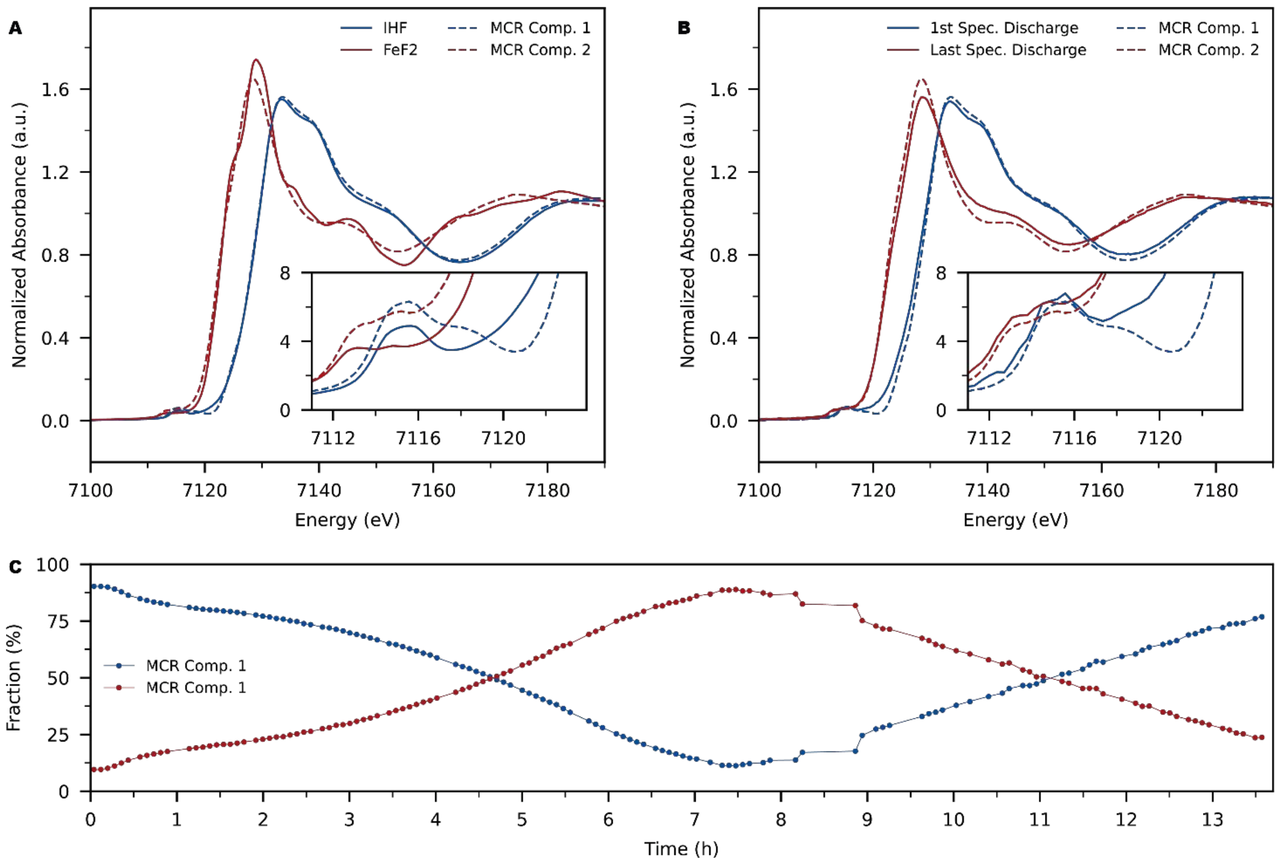

Figure S16. MCR ALS analysis for the *operando* XAS spectra of full cells with Pyr-IHF containing cathodes. **A**) shows the Pyr-IHF and FeF_2 reference spectra used for initial guesses of the two components together with the extracted MCR components. **B**) shows the first and last acquired spectrum during initial discharge together with the extracted MCR components and **C**) shows the concentration profiles of the two components during the first cycle. The R^2 value for the fit was 99.9978%.

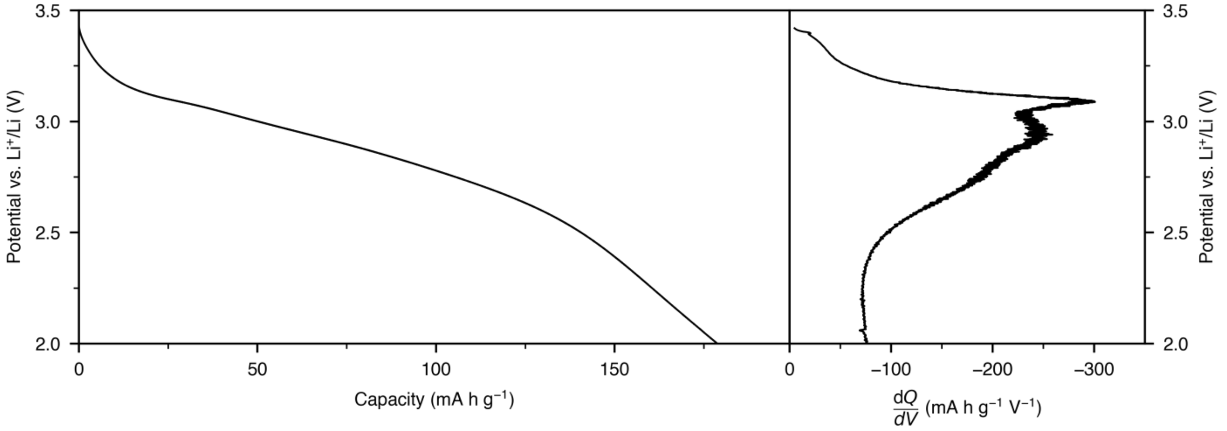

Figure S17. Voltage profile and dQ/dV plot of the initial discharge of the full cell with Pyr-IHF cathode presented in Figure 3d.

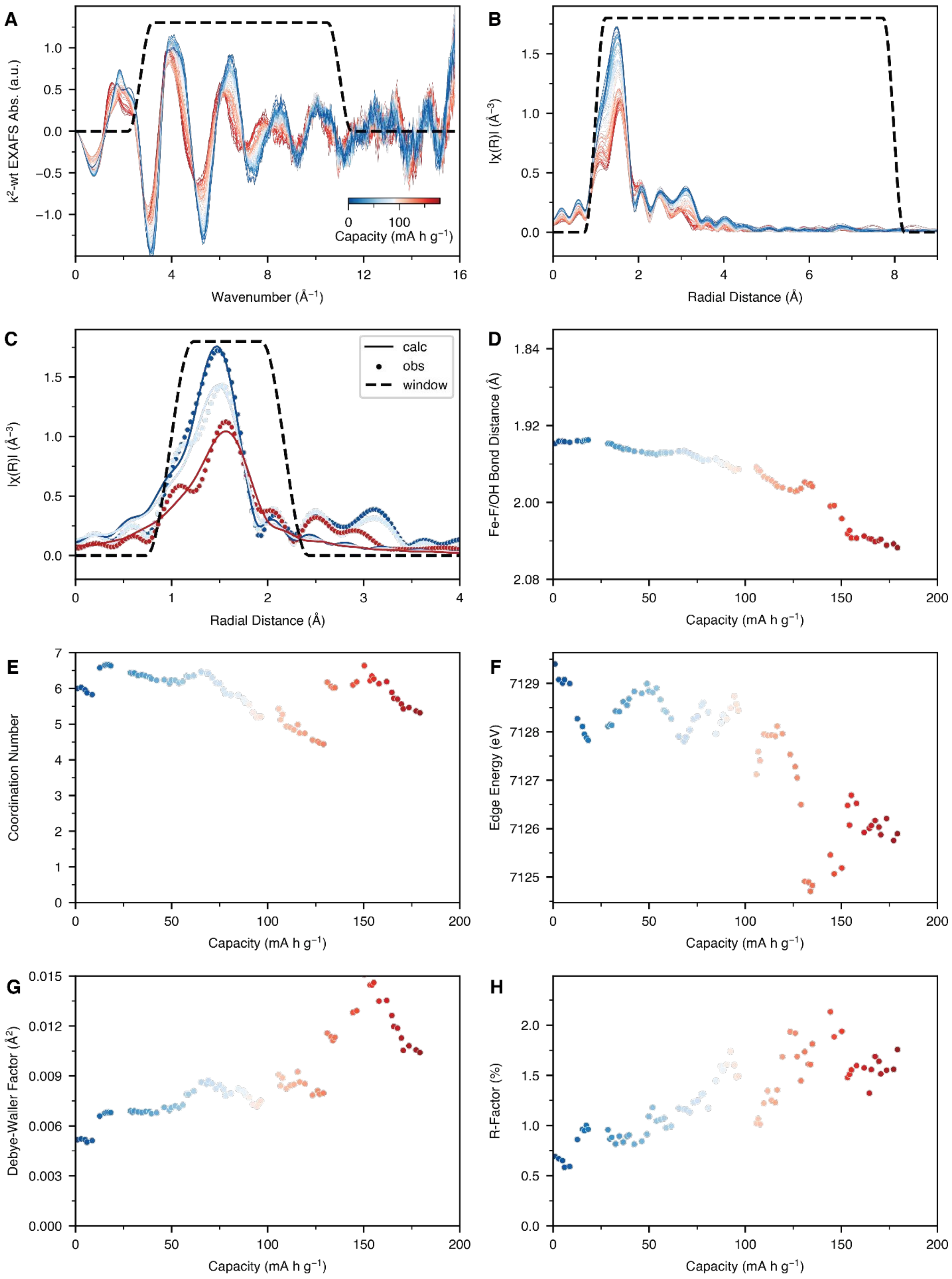
A
B
C
D
E
F
G
H
k^2-wt EXAFS Abs. (a.u.)
Wavenumber (Å^{-1})
Capacity (mA h g^{-1})
|χ(R)| (Å^{-3})
Radial Distance (Å)
calc
obs
window
Fe-F/OH Bond Distance (Å)
Coordination Number
Edge Energy (eV)
Debye-Waller Factor (Å^2)
R-Factor (%)

Figure S18. EXAFS analysis for the *operando* XAS spectra of full cells with Pyr-IHF containing cathodes. **A**) shows the k^2-weighted EXAFS signal in *k*-space (**A**) and *r*-space (**B**). Color corresponds to the electrochemical capacity. The black dashed lines indicate the windows used for the forwards and backwards Fourier transformation. **C**) shows the first-shell EXAFS fit of three selected spectra. **D-H**) Results of the EXAFS fit, showing the Fe-F/OH bond distance (**D**), coordination number (**E**), edge energy (**F**), Debye-Waller factor (**G**), and *R*-factors (**H**) for all the fitted spectra.

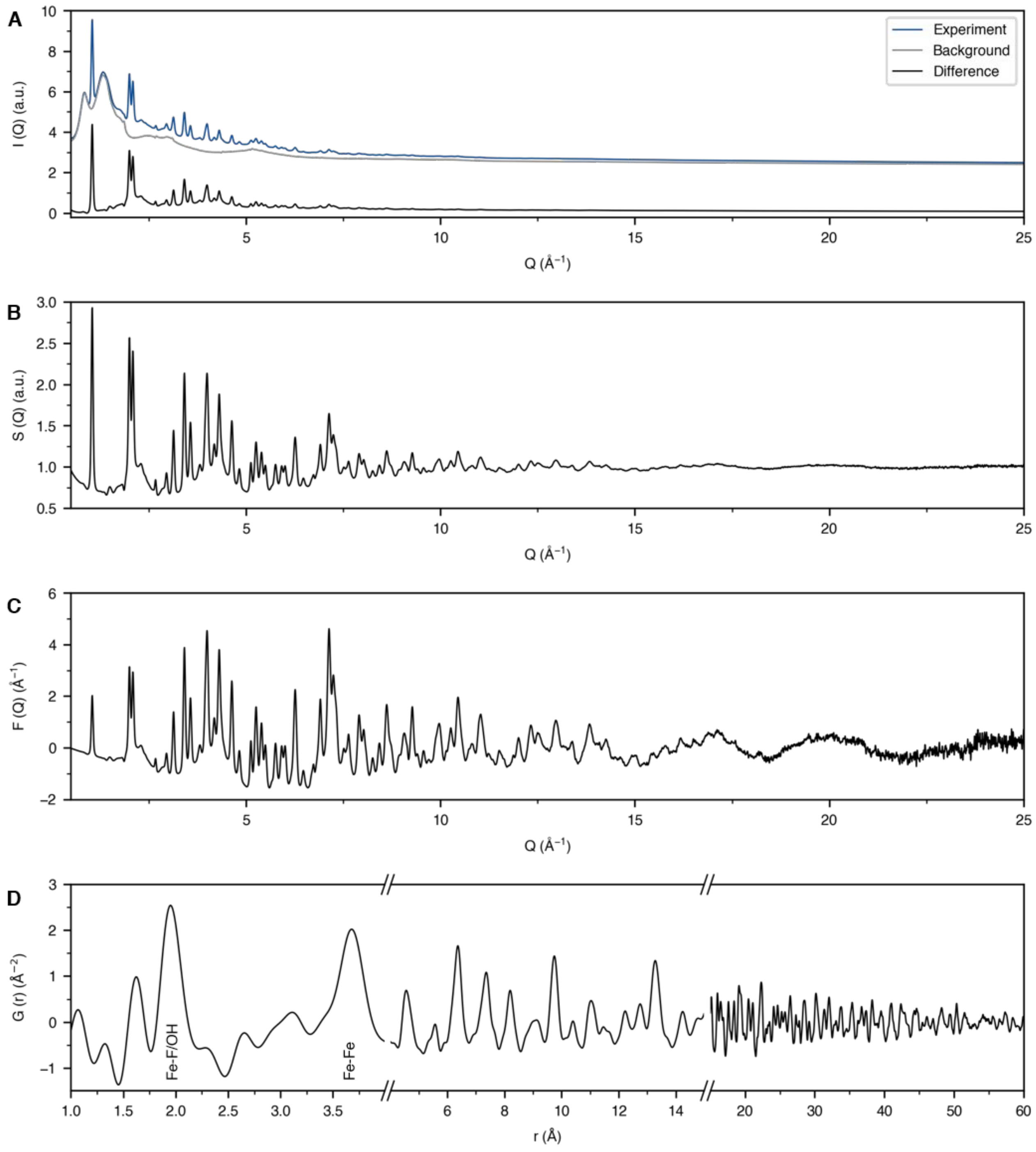

Figure S19. Synchrotron X-ray total scattering of pristine cathodes containing heat-treated Pyr-IHF after cell assembly and equilibration. The difference between raw data and background was used for subsequent analysis.

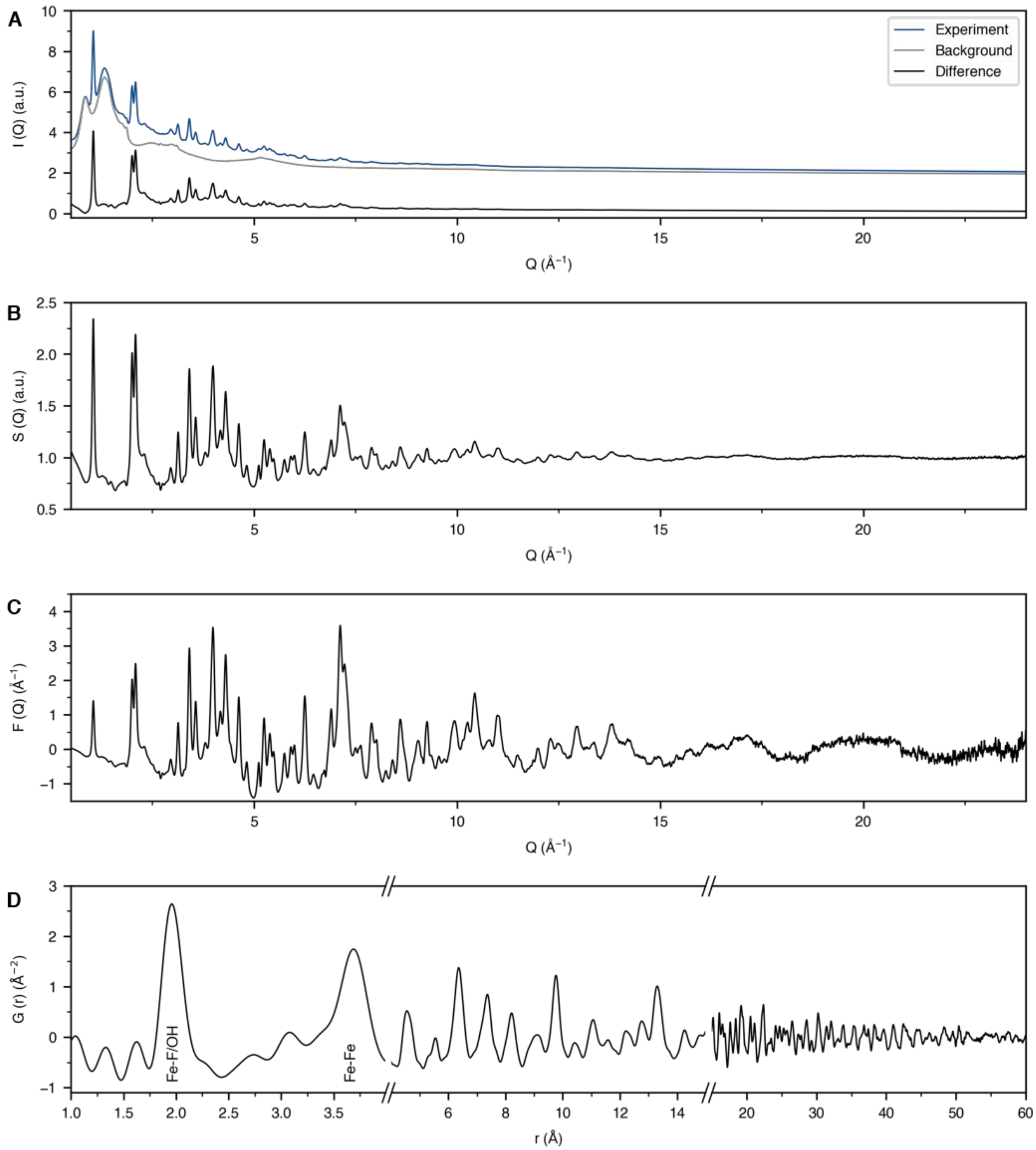

Figure S20. Synchrotron X-ray total scattering of cathodes containing heat-treated Pyr-IHF discharged to 3 V. The difference between raw data and background was used for subsequent analysis.

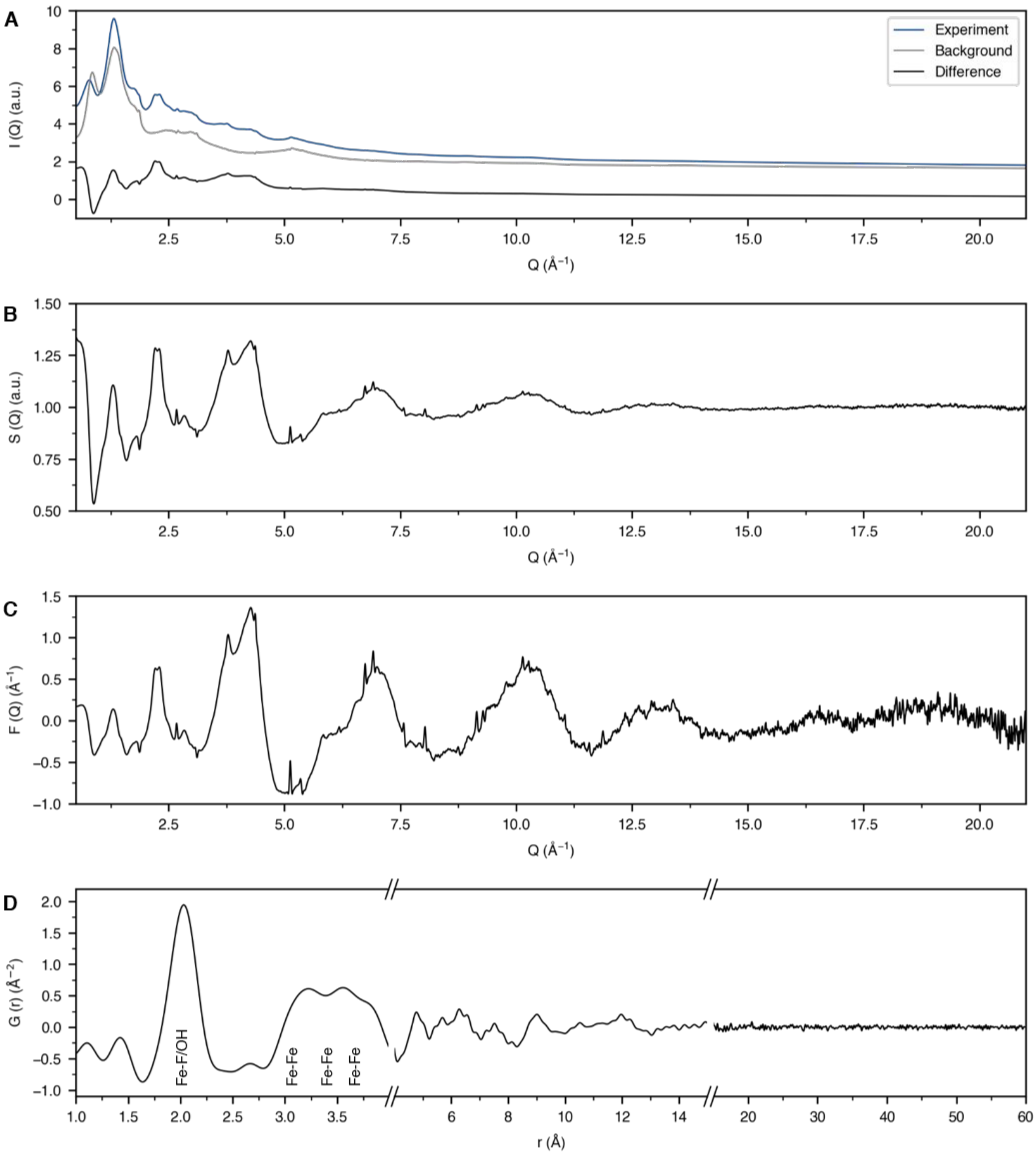

Figure S21. Synchrotron X-ray total scattering of cathodes containing heat-treated Pyr-IHF discharged to 2.5 V. The difference between raw data and background was used for subsequent analysis. The minor crystalline impurity is due to residual Al current collector from scraping off the cathode and placing it inside the capillary for measurement.

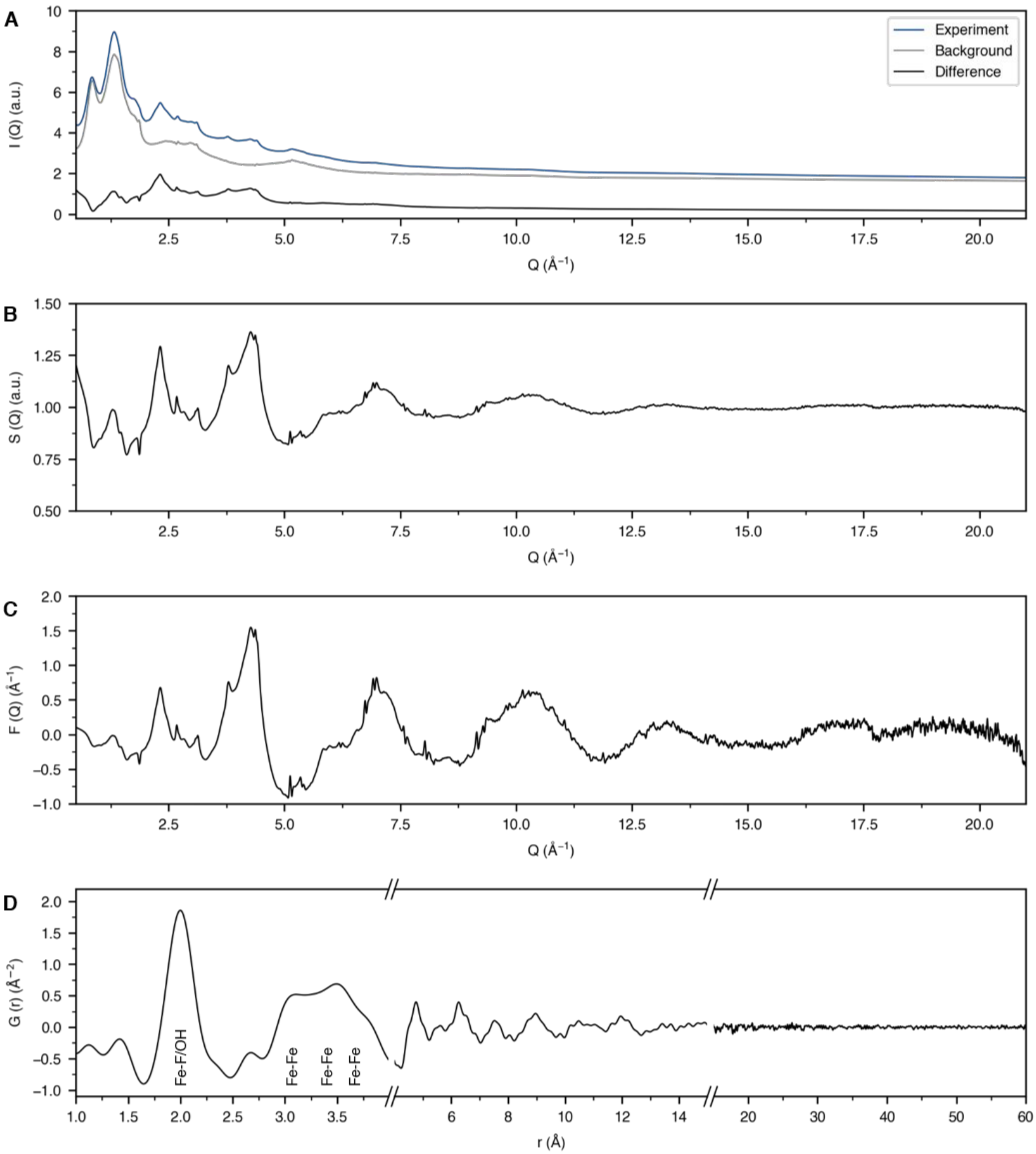

Figure S22. Synchrotron X-ray total scattering of cathodes containing heat-treated Pyr-IHF discharged to 2 V. The difference between raw data and background was used for subsequent analysis. The minor crystalline impurity is due to residual Al current collector from scraping off the cathode and placing it inside the capillary for measurement.

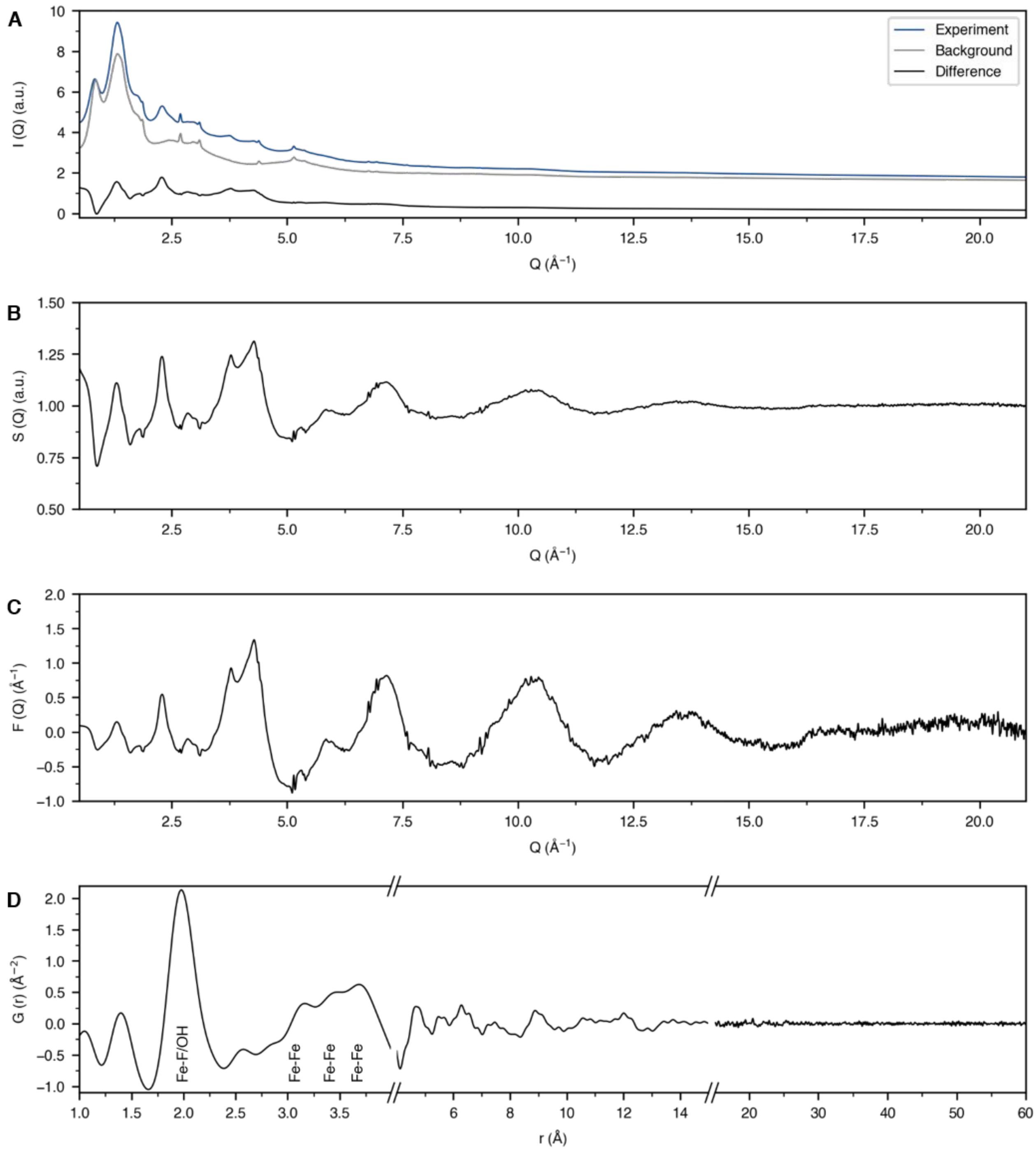

Figure S23. Synchrotron X-ray total scattering of cathodes containing heat-treated Pyr-IHF discharged to 2 V and recharged to 3.5 V. The difference between raw data and background was used for subsequent analysis. The minor crystalline impurity is due to residual Al current collector from scraping off the cathode and placing it inside the capillary for measurement.

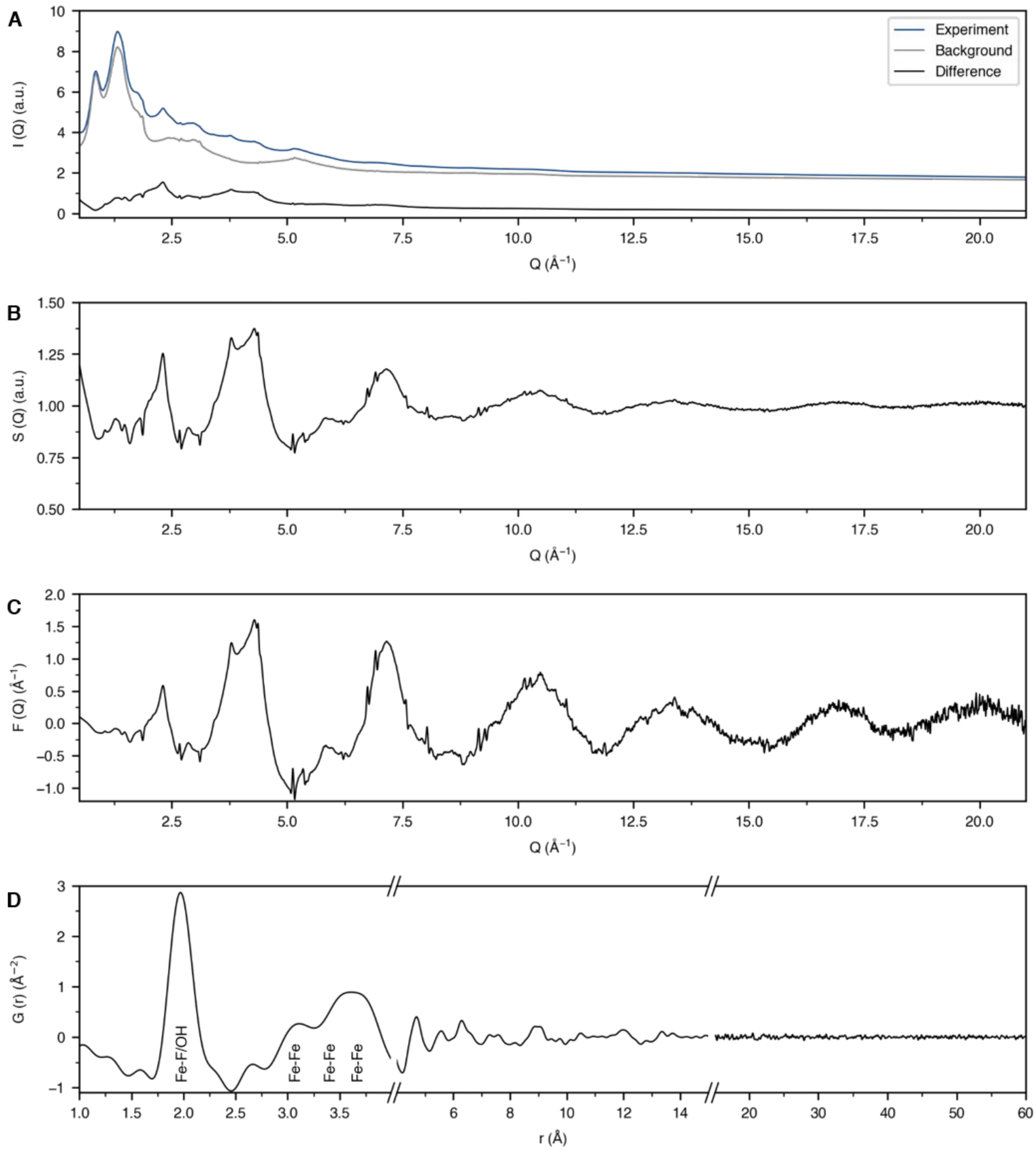

Figure S24. Synchrotron X-ray total scattering of cathodes containing heat-treated Pyr-IHF discharged to 2 V and recharged to 4.2 V. The difference between raw data and background was used for subsequent analysis. The minor crystalline impurity is due to residual Al current collector from scraping off the cathode and placing it inside the capillary for measurement.

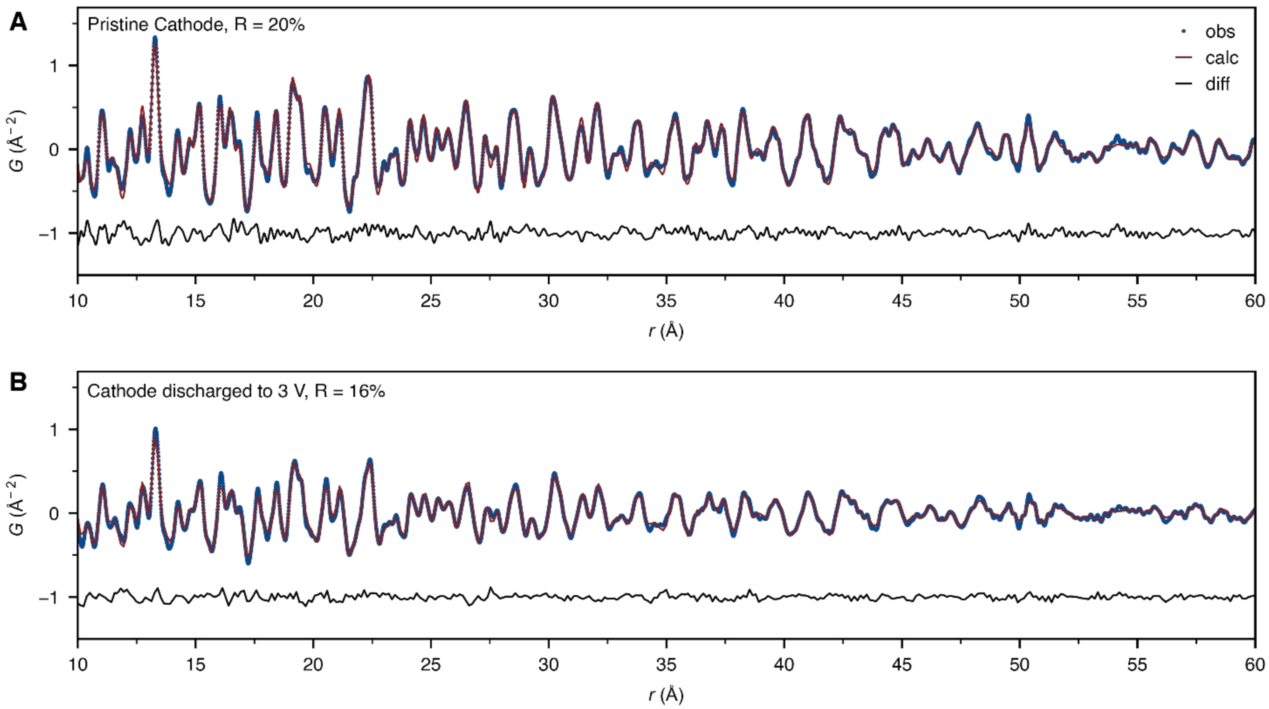

Figure S25. Refinement of the synchrotron PDF of pristine Pyr-IHF cathodes (**A**) and Pyr-IHF cathodes discharged to 3 V (**B**).

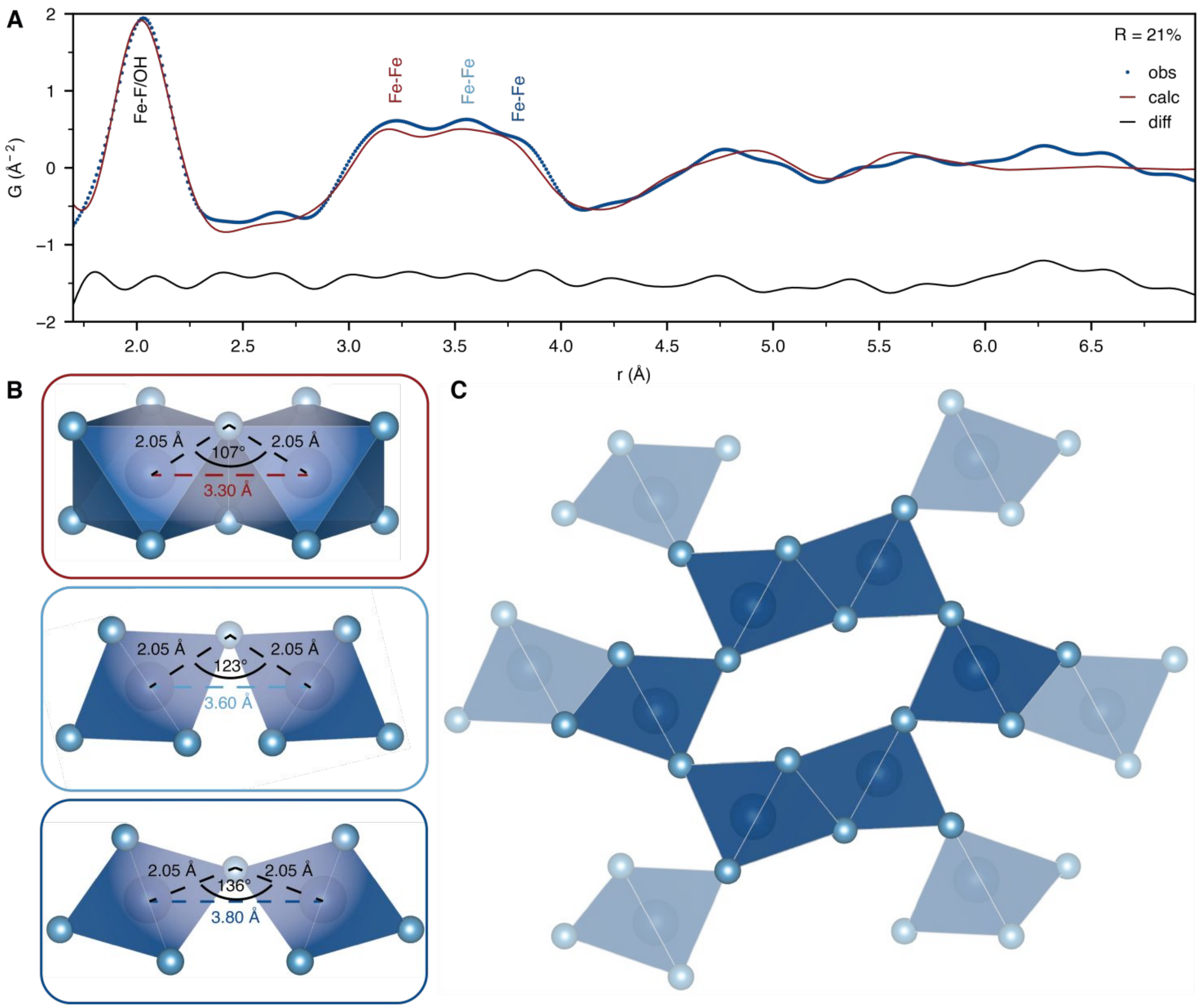

Figure S26. Refinement of the synchrotron PDF of Pyr-IHF cathodes discharged to 2.5 V (**A**), model-free bond angle analysis (**B**), and a proposed model for the channel structure compatible with the PDF refinement (**C**). The refined values are summarized in Table S9.

Table S9. Overview of the refined parameters from PDF for Pyr-IHF cathodes discharged to 2.5 V. Values in brackets indicate estimated standard error. If no error is given, the parameter was not refined.

S.G Pnma[19]				$d_{Particle}$ = 7.5(1.5) Å			
a = 9.7(4) Å		b = 3.15(5) Å		c = 4.58(13) Å		α = β = γ = 90°	
Site	Wyck.	x	y	y	Occ.	U_{iso} (Å^2)	
Fe1	4c	0.859(4)	¼	0.10(3)	1	0.010(12)	
F1	4c	0.063(24)	¼	0.17(4)	1	0.016	
F2	4c	0.802(18)	¾	0.36(6)	1	0.016	
No. parameters = 13			χ^2 = 5.8			χ^2_{red} = 0.011	R_w = 20.7%

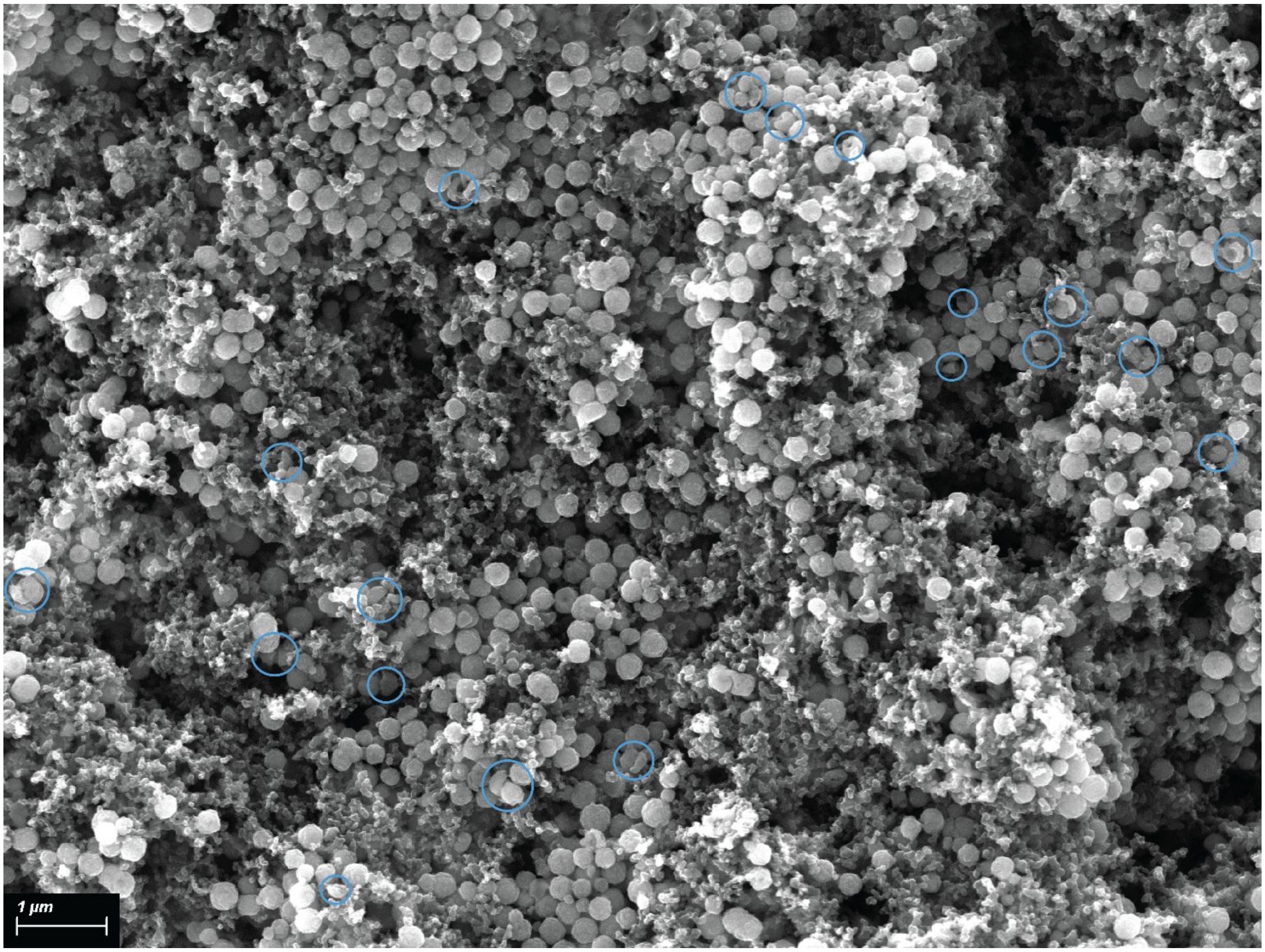

Figure S27. SEM micrographs of pristine Pyr-IHF cathodes used for the determining the fraction of cracked particles (N_{tot} = 934). Blue circles indicate cracked particles

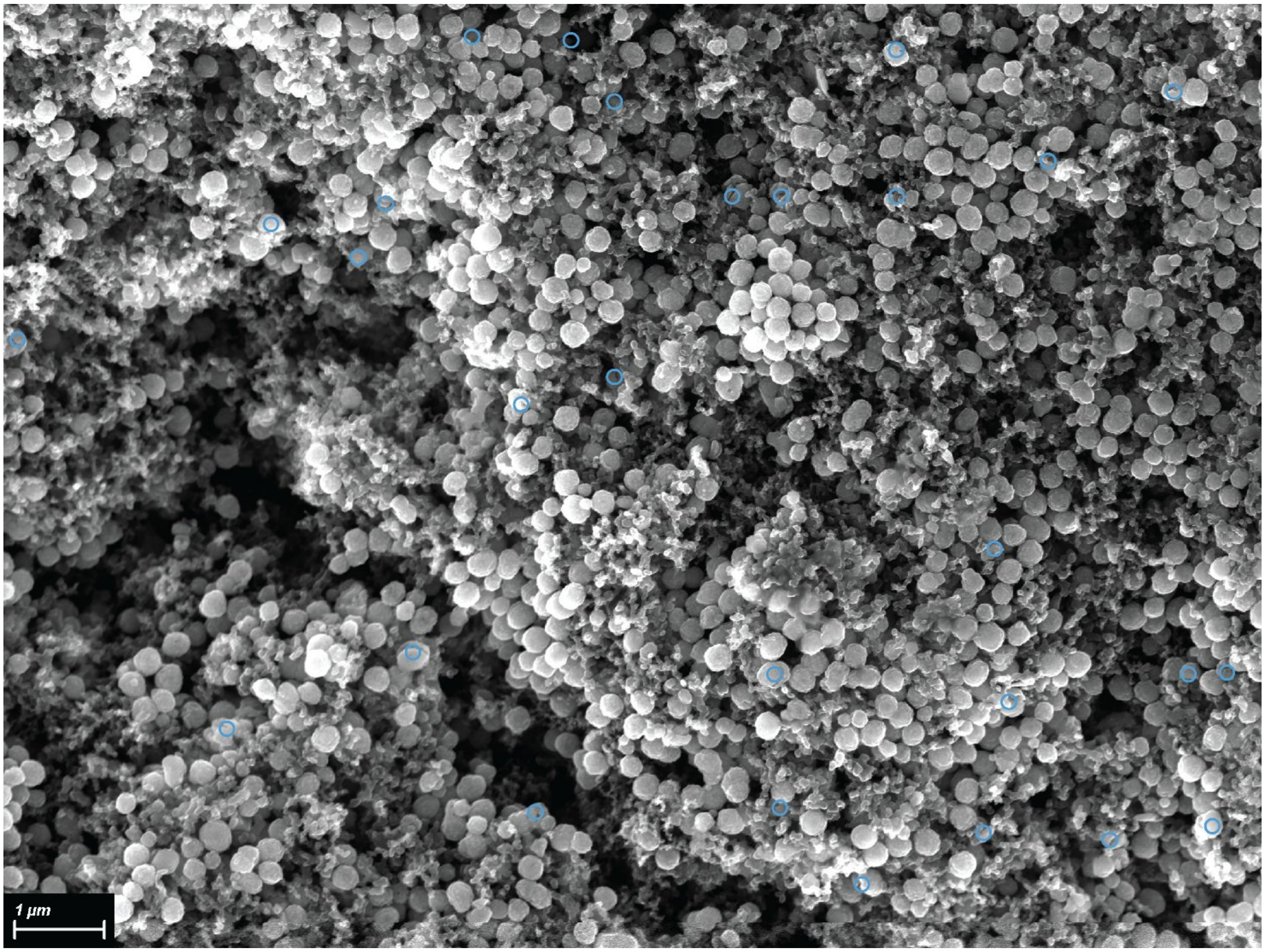

Figure S28. SEM micrographs of Pyr-IHF cathodes discharged to 3 V *vs.* Li^{+}/Li used for the determining the fraction of cracked particles (N_{tot} = 996). Blue circles indicate cracked particles

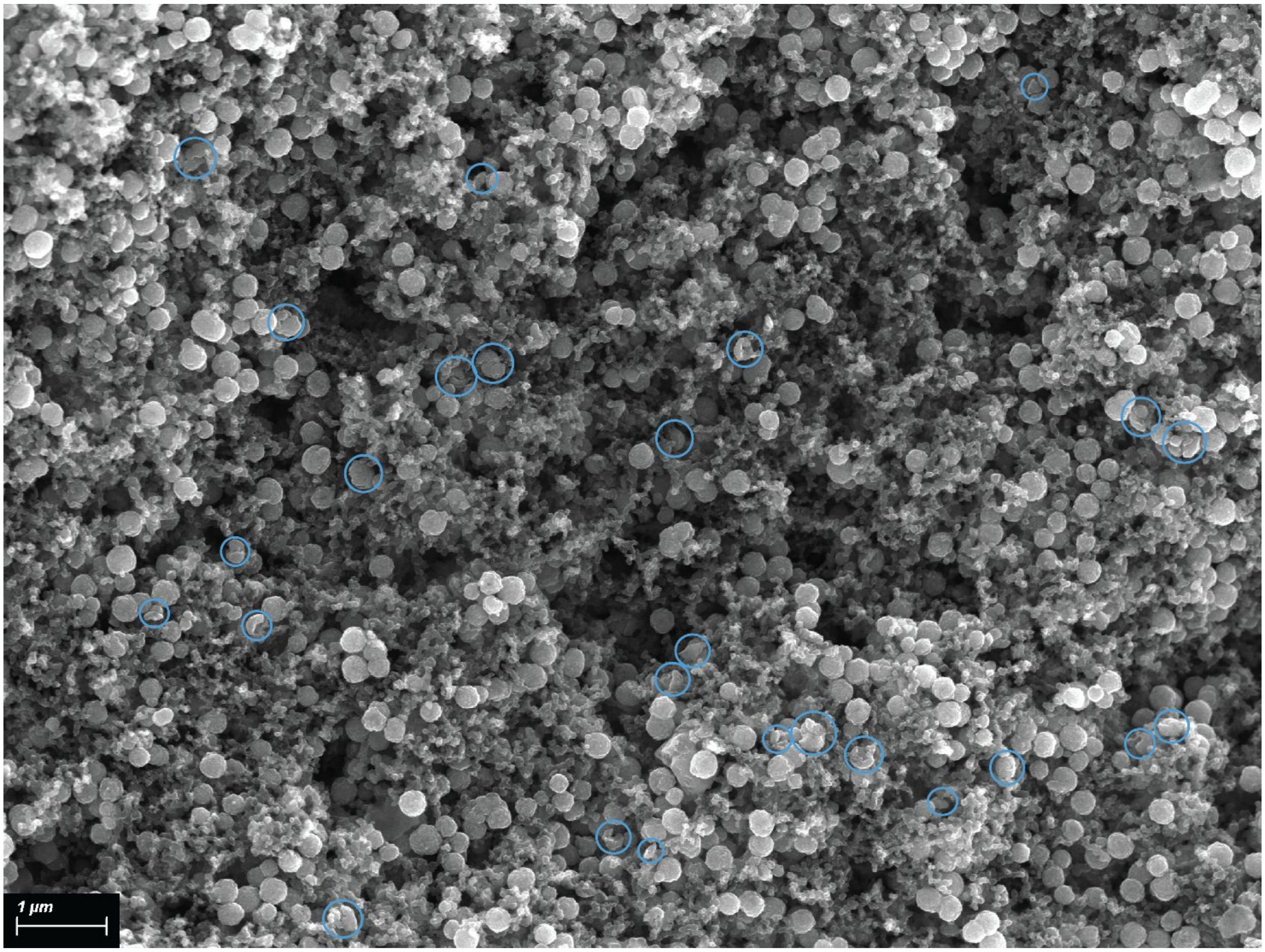

Figure S29. SEM micrographs of Pyr-IHF cathodes discharged to 2 V *vs.* Li^{+}/Li used for the determining the fraction of cracked particles (N_{tot} = 875). Blue circles indicate cracked particles

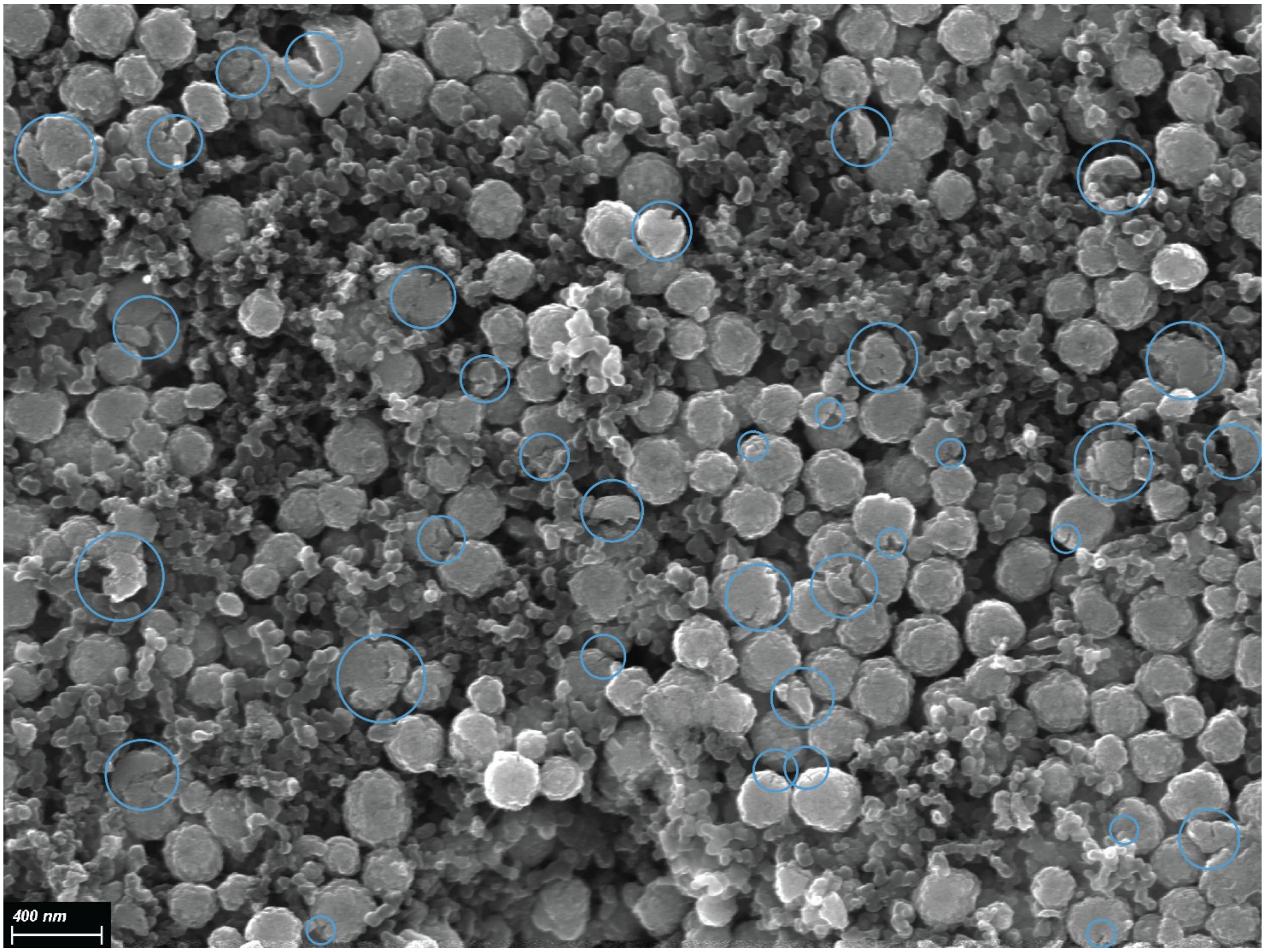

Figure S30. SEM micrographs of Pyr-IHF cathodes cycled for 100 cycles (2 – 4.2 V vs. Li^+/Li), used for the determining the fraction of cracked particles (N_{tot} = 205). Blue circles indicate cracked particles

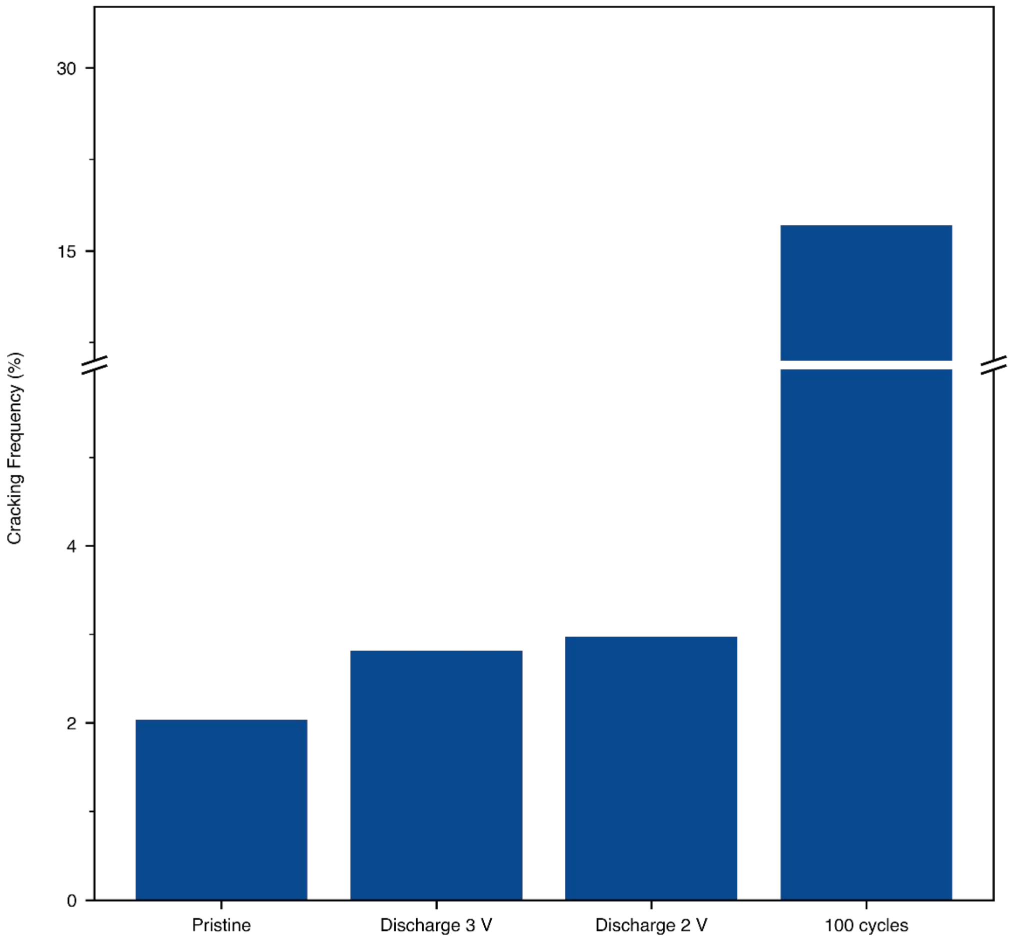

Figure S31. Cracking frequency of Pyr-IHF particles during different stages of charge and discharge, as determined from the SEM micrographs (Figures S27 to S30).

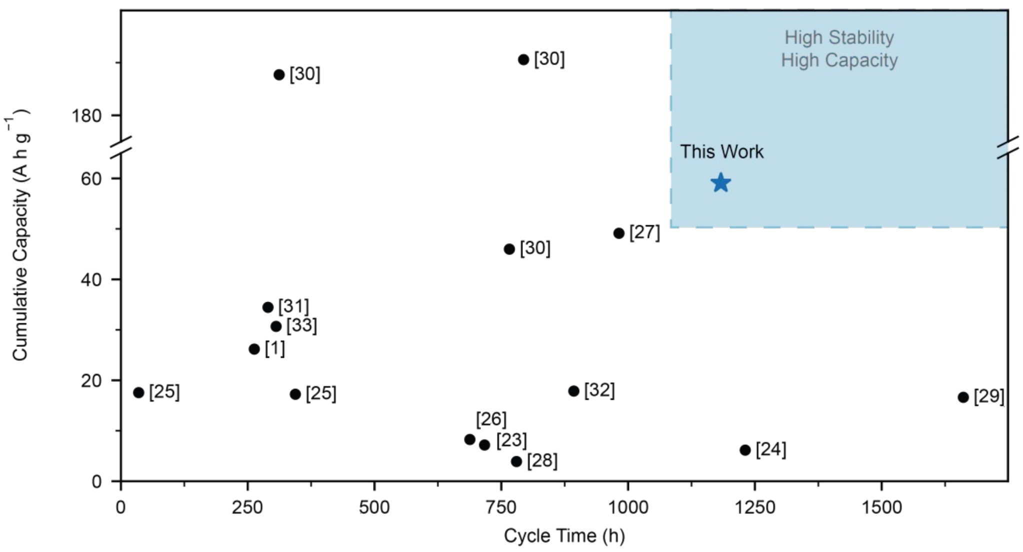

Figure S32. Overview of cycle time vs. cumulative discharge capacity of iron (oxy/hydroxy) fluorides (lower cutoff voltage 2 – 4.x V *vs.* Li^+/Li) reported in the literature,[1, 23-33] as well as in this work.

REFERENCES

(1) Baumgärtner, J. F.; Wörle, M.; Guntlin, C. P.; Krumeich, F.; Siegrist, S.; Vogt, V.; Stoian, D. C.; Chernyshov, D.; van Beek, W.; Kravchyk, K. V.; et al. Pyrochlore-Type Iron Hydroxy Fluorides as Low-Cost Lithium-Ion Cathode Materials for Stationary Energy Storage. *Adv. Mater.* **2023**, *35* (49), 2304158. DOI: 10.1002/adma.202304158.

(2) Abdolhosseinzadeh, S.; Zhang, C.; Schneider, R.; Shakoorioskooie, M.; Nüesch, F.; Heier, J. A Universal Approach for Room-Temperature Printing and Coating of 2D Materials. *Adv. Mater.* **2022**, *34* (4), 2103660. DOI: 10.1002/adma.202103660.

(3) Dyadkin, V.; Pattison, P.; Dmitriev, V.; Chernyshov, D. A new multipurpose diffractometer PILATUS@SNBL. *J. Synchrotron Rad.* **2016**, *23* (3), 825-829. DOI: 10.1107/S1600577516002411.

(4) Toby, B. H.; Von Dreele, R. B. GSAS-II : the genesis of a modern open-source all purpose crystallography software package. *J. Appl. Crystallogr.* **2013**, *46* (2), 544-549. DOI: 10.1107/S0021889813003531.

(5) Juhas, P.; Davis, T.; Farrow, C. L.; Billinge, S. J. L. PDFgetX3: a rapid and highly automatable program for processing powder diffraction data into total scattering pair distribution functions. *J. Appl. Crystallogr.* **2013**, *46* (2), 560-566. DOI: 10.1107/S0021889813005190.

(6) Farrow, C. L.; Juhas, P.; Liu, J. W.; Bryndin, D.; Božin, E. S.; Bloch, J.; Proffen, T.; Billinge, S. J. L. PDFfit2 and PDFgui: computer programs for studying nanostructure in crystals. *J. Condens. Matter Phys.* **2007**, *19* (33), 335219. DOI: 10.1088/0953-8984/19/33/335219.

(7) Juhas, P.; Farrow, C. L.; Yang, X.; Knox, K. R.; Billinge, S. J. L. Complex modeling: a strategy and software program for combining multiple information sources to solve ill posed structure and nanostructure inverse problems. *Acta Cryst. A* **2015**, *71* (6), 562-568. DOI: 10.1107/S2053273315014473.

(8) Bleith, P.; van Beek, W.; Kaiser, H.; Novák, P.; Villevieille, C. Simultaneous in Situ X-ray Absorption Spectroscopy and X-ray Diffraction Studies on Battery Materials: The Case of $Fe_{0.5}TiOPO_4$. *J. Phys. Chem. C* **2015**, *119* (7), 3466-3471. DOI: 10.1021/jp511042x.

(9) Ravel, B.; Newville, M. ATHENA, ARTEMIS, HEPHAESTUS: data analysis for X-ray absorption spectroscopy using IFEFFIT. *J. Synchrotron Rad.* **2005**, *12* (4), 537-541. DOI: 10.1107/S0909049505012719.

(10) Newville, M. Larch: An Analysis Package for XAFS and Related Spectroscopies. *J. Phys. Conf. Ser.* **2013**, *430* (1), 012007. DOI: 10.1088/1742-6596/430/1/012007.

(11) Figueroa, S. J. A.; Prestipino, C. PrestoPronto: a code devoted to handling large data sets. *J. Phys. Conf. Ser.* **2016**, *712* (1), 012012. DOI: 10.1088/1742-6596/712/1/012012.

(12) Kaiser, H. F. The Application of Electronic Computers to Factor Analysis. *Educ. Psychol. Meas.* **1960**, *20* (1), 141-151. DOI: 10.1177/001316446002000116.

(13) de Juan, A.; Jaumot, J.; Tauler, R. Multivariate Curve Resolution (MCR). Solving the mixture analysis problem. *Anal. Methods* **2014**, *6* (14), 4964-4976, 10.1039/C4AY00571F. DOI: 10.1039/C4AY00571F.

(14) Schneider, C. A.; Rasband, W. S.; Eliceiri, K. W. NIH Image to ImageJ: 25 years of image analysis. *Nat. Methods* **2012**, *9* (7), 671-675. DOI: 10.1038/nmeth.2089.

(15) *A Python library for reliability engineering*; Zenodo: 2023. (accessed 11.04.2024).

(16) Baumgärtner, J. F.; Krumeich, F.; Wörle, M.; Kravchyk, K. V.; Kovalenko, M. V. Thermal synthesis of conversion-type bismuth fluoride cathodes for high-energy-density Li-ion batteries. *Commun. Chem.* **2022**, *5* (1), 1-8. DOI: 10.1038/s42004-021-00622-y.
(17) Li, C.; Mu, X.; van Aken, P. A.; Maier, J. A High-Capacity Cathode for Lithium Batteries Consisting of Porous Microspheres of Highly Amorphized Iron Fluoride Densified from Its Open Parent Phase. *Adv. Energy Mater.* **2013**, *3* (1), 113-119. DOI: 10.1002/aenm.201200209.
(18) Baur, W. H.; Khan, A. A. Rutile-type compounds. IV. SiO2, GeO2 and a comparison with other rutile-type structures. *Acta Crystallographica Section B* **1971**, *27* (11), 2133-2139. DOI: 10.1107/S0567740871005466.
(19) Ben Yahia, H.; Shikano, M.; Tabuchi, M.; Kobayashi, H.; Avdeev, M.; Tan, T. T.; Liu, S.; Ling, C. D. Synthesis and Characterization of the Crystal and Magnetic Structures and Properties of the Hydroxyfluorides Fe(OH)F and Co(OH)F. *Inorg. Chem.* **2014**, *53* (1), 365-374. DOI: 10.1021/ic402294g.
(20) Leblanc, M.; Ferey, G.; Chevallier, P.; Calage, Y.; De Pape, R. Hexagonal tungsten bronze-type FeIII fluoride: (H2O)0.33FeF3; crystal structure, magnetic properties, dehydration to a new form of iron trifluoride. *Journal of Solid State Chemistry* **1983**, *47* (1), 53-58. DOI: 10.1016/0022-4596(83)90041-5.
(21) Hepworth, M. A.; Jack, K. H.; Peacock, R. D.; Westland, G. J. The crystal structures of the trifluorides of iron, cobalt, ruthenium, rhodium, palladium and iridium. *Acta Cryst.* **1957**, *10* (1), 63-69. DOI: 10.1107/S0365110X57000158.
(22) Zepeda-Alarcon, E.; Nakotte, H.; Gualtieri, A. F.; King, G.; Page, K.; Vogel, S. C.; Wang, H.-W.; Wenk, H.-R. Magnetic and nuclear structure of goethite ([alpha]-FeOOH): a neutron diffraction study. *J. Appl. Crystallogr.* **2014**, *47* (6), 1983-1991. DOI: 10.1107/S1600576714022651.
(23) Ma, D.-l.; Cao, Z.-y.; Wang, H.-g.; Huang, X.-l.; Wang, L.-m.; Zhang, X.-b. Three-dimensionally ordered macroporous FeF3 and its in situ homogenous polymerization coating for high energy and power density lithium ion batteries. *Energy Environ. Sci.* **2012**, *5* (9), 8538. DOI: 10.1039/c2ee22568a.
(24) Shi, Y.-L.; Wu, N.; Shen, M.-F.; Cui, Y.-L.; Jiang, L.; Qiang, Y.-H.; Zhuang, Q.-C. Electrochemical Behavior of Iron(III) Fluoride Trihydrate as a Cathode in Lithium-Ion Batteries. *ChemElectroChem* **2014**, *1* (3), 645-654. DOI: 10.1002/celc.201300069.
(25) Guntlin, C. P.; Zünd, T.; Kravchyk, K. V.; Wörle, M.; Bodnarchuk, M. I.; Kovalenko, M. V. Nanocrystalline FeF3 and MF2 (M = Fe, Co, and Mn) from metal trifluoroacetates and their Li(Na)-ion storage properties. *J. Mater. Chem. A* **2017**, *5* (16), 7383-7393. DOI: 10.1039/C7TA00862G.
(26) Bai, Y.; Zhou, X.; Zhan, C.; Ma, L.; Yuan, Y.; Wu, C.; Chen, M.; Chen, G.; Ni, Q.; Wu, F.; et al. 3D Hierarchical nano-flake/micro-flower iron fluoride with hydration water induced tunnels for secondary lithium battery cathodes. *Nano Energy* **2017**, *32*, 10-18. DOI: 10.1016/j.nanoen.2016.12.017.
(27) Zhao, E.; Borodin, O.; Gao, X.; Lei, D.; Xiao, Y.; Ren, X.; Fu, W.; Magasinski, A.; Turcheniuk, K.; Yushin, G. Lithium–Iron (III) Fluoride Battery with Double Surface Protection. *Adv. Energy Mater.* **2018**, *8* (26), 1800721. DOI: 10.1002/aenm.201800721.
(28) Tawa, S.; Sato, Y.; Orikasa, Y.; Matsumoto, K.; Hagiwara, R. Lithium fluoride/iron difluoride composite prepared by a fluorolytic sol–gel method: Its electrochemical behavior and charge–discharge mechanism as a cathode material for lithium secondary batteries. *J. Power Sources* **2019**, *412*, 180-188. DOI: 10.1016/j.jpowsour.2018.11.046.

(29) Chen, G.; Zhou, X.; Bai, Y.; Yuan, Y.; Li, Y.; Chen, M.; Ma, L.; Tan, G.; Hu, J.; Wang, Z.; et al. Enhanced lithium storage capability of FeF3·0.33 H2O single crystal with active insertion site exposed. *Nano Energy* **2019**, *56*, 884-892. DOI: 10.1016/j.nanoen.2018.11.080.
(30) Wu, F.; Srot, V.; Chen, S.; Lorger, S.; van Aken, P. A.; Maier, J.; Yu, Y. 3D Honeycomb Architecture Enables a High-Rate and Long-Life Iron (III) Fluoride-Lithium Battery. *Adv. Mater.* **2019**, *31* (43), 1905146. DOI: 10.1002/adma.201905146.
(31) Zhang, L.; Yu, L.; Li, O. L.; Choi, S.-Y.; Saeed, G.; Kim, K. H. FeF3·0.33 H2O@carbon nanosheets with honeycomb architectures for high-capacity lithium-ion cathode storage by enhanced pseudocapacitance. *J. Mater. Chem. A* **2021**, *9* (30), 16370-16383. DOI: 10.1039/D1TA03141D.
(32) Ding, J.; Zhou, X.; Luo, C.; Yang, J.; Tang, J. Bamboo-structured N-doped CNTs/FeF3·0.33 H2O derived from melamine as a high-performance cathode for Li-ion batteries. *New. J. Chem.* **2021**, *45* (38), 18019-18024. DOI: 10.1039/D1NJ02855C.
(33) Ding, J.; Zhou, X.; Luo, C.; Xu, H.; Yang, J.; Tang, J. In situ synthesis of graphene-like N, S co-doped carbon nanosheets/FeF3·0.33 H2O composite as cathode material for Li-ion battery. *J. Mater. Sci.* **2022**, *57* (2), 1261-1270. DOI: 10.1007/s10853-021-06625-3.
